# Synergistic Dual Slip‐Link Toughening of a Water‐Rich Double Network Hydrogel Combining Slide‐Ring and Highly Entangled Networks

**DOI:** 10.1002/advs.75525

**Published:** 2026-05-05

**Authors:** Subhankar Mandal, Saleh Assadi, Aseem Milind Visal, Ignacio Lorente Montero, Franck J. Vernerey, Carson J. Bruns

**Affiliations:** ^1^ ATLAS Institute University of Colorado Boulder Colorado USA; ^2^ Paul M. Rady Department of Mechanical Engineering University of Colorado Boulder Colorado USA; ^3^ Materials Science and Engineering University of Colorado Boulder Colorado USA

**Keywords:** double network, high entanglement, hydrogel, slide‐ring gel

## Abstract

Slide‐ring network (SRN) hydrogels derived from ring‐crosslinked polyrotaxanes exhibit exceptional mechanical properties attributable to a pulley effect, whereby mobile‐ring crosslinks redistribute tension under deformation through a slip‐link mechanism. However, SRN hydrogels weaken severely upon swelling in water, limiting their utility at high water content (>90 wt.%). Here, two distinct physical slip‐link mechanisms are combined in a highly entangled slide‐ring double network (HESRDN) hydrogel: the pulley effect of a polyrotaxane slide‐ring network and the entangled chains of a sparsely cross‐linked polyacrylamide network. HESRDN is prepared by photopolymerization of acrylamide/*N*,*N*'‐methylenebis(acrylamide) within a partially swollen slide‐ring hydrogel. The dual slip‐link architecture synergistically strengthens and toughens the hydrogel far beyond the sum of the component networks, yielding high work of fracture (1275 kJm^−3^), toughness (2020 Jm^−2^), and near‐complete reversibility (99.7%) at >91 wt.% water. HESRDN withstands continuous friction for over 12 h without rupture, compared to minutes for the SRN and 5 h for the HEN component networks, reflecting the unique capacity of the dual slip‐link architecture to delocalize and redistribute stress under sustained mechanical loading.

## Graphical abstract

Two distinct slip‐link mechanisms – the slide‐ring pulley effect and highly entangled chains – are combined in a water‐rich double network hydrogel, achieving synergistic toughening, near‐complete reversibility, and exceptional friction tear resistance at >91 wt.% water.

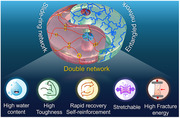

## 1 Introduction

Hydrogels are three‐dimensional polymeric structures that contain a substantial amount of water, presenting considerable opportunities [1] for applications in diverse areas such as tissue engineering [2], drug delivery [3], soft actuators [4], and various load‐bearing applications [5]. In general, hydrogels are often limited by lower mechanical strength at higher water content, inferior to natural tissues such as cartilage, which exhibits high stiffness, toughness, and fatigue resistance [6, 7]. Most of the toughest hydrogels reported in the literature are formulated below 90 wt.% water, above which it becomes especially challenging to achieve high strength, extensibility, toughness, and rapid recovery simultaneously (see Table 1). Maximizing water content is broadly desirable; since the polymer component is invariably far more costly and resource‐intensive than water, higher water content directly reduces material cost and environmental burden. Moreover, higher water content expands the aqueous volume available for ion transport and molecular diffusion (critical for drug delivery, hydrogel electrodes, and ion‐transport membranes) since solute diffusivity scales inversely with polymer volume fraction [8]. The development of more mechanically robust hydrogels at >90 wt.% water is thus a scientific challenge and a practical imperative.

**TABLE 1:** Comparison of mechanical properties among different single and double‐network (DN) hydrogels incorporating slip‐link motifs.

Hydrogel	WC^a^ (%)	Γ ^b^ (${\mathrm{Jm}}^{-2}$)	U ^c^ (${\mathrm{kJm}}^{-3}$)	E ^d^ (kPa)	$U_{2}/U_{1}$ ^e^ (%)	Ref.
**Single network**						
Highly entangled PEG	92	1575	854	675	99	[38]
Highly entangled PAAm gel	90	1450	500	150	96	[54]
Hydrophilic/phobic PAAm	86	26000	18800	357	99	[17]
Slide‐Ring (SR) polyrotaxane gel	51	3600	22000	150	99	[44]
**Traditional DN**						
PAAm‐PAMPS DN	90	1000‐4500	11000	100‐1000	32	[9]
Alginate‐${\mathrm{Ca}}^{2+}$/ PAAm	90	9000	2500	29	22	[6]
Agar/HPAAm DN gel	85	1000	1023	106	20	[22]
Alginate/ PVA polyelectrolyte	70	1450	5430	540	75	[28]
Agar‐PAAm DN gel	84	3960	15370	447	61	[23]
**Highly entangled DN**						
Polyprotein/ PAAm	90	870	200	6	95	[37]
Highly Entangled PAAm‐AMPS	89	8340	2490	180	99	[55]
**Slide‐Ring DN**						
Polymerizable rotaxane/ PAAm	60	900	270	12	97.5	[49]
αCD/ PAA‐co‐PAAm‐${\mathrm{Fe}}^{3+}$	83	10000	7200	9.5	55	[46]
NIPAM‐NaAAc‐HPαCD	80	—	—	8.3	—	[50]
PEG‐HPαCD/PAAm	71	—	17400	35.6	97	[51]
Modified Pseudorotaxane/PAAm	75	—	35840	—	28	[52]
Substituted PR‐PAAm/H‐bond	50	—	1100	156	92	[53]
**Highly Entangled Slide‐Ring DN**						
SRN/PAAm HESRDN (This Work)	91	2020	1275	202	99.7	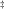

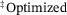
 highly entangled slide ring double network sample developed in the current work.

a Water content of the hydrogel.

b Toughness.

c Work of fracture.

d Stiffness.

e Reversibility in hysteresis loading‐unloading cycles.

Emerging strategies [9] such as double networking [10, 11, 12], dual cross‐linking [13, 14, 15], hydrophobic interactions [16, 17, 18], nanocomposite reinforcing [17, 19, 20], ion‐pairing [21], and combinations thereof [22, 23, 24, 25] have been shown to strengthen hydrogels dramatically. For example, double network (DN) hydrogels based on polyacrylamide (PAAm) have been in development for two decades, exhibiting improved toughness through various modes of destructive and non‐destructive energy dissipation afforded by the interpenetrating second network [12, 26]. A PAAm network strengthened by a poly(2‐acrylamido‐2‐methyl‐1‐propanesulfonic acid) (PAMPS) network leads to compressive strengths up to 40 MPa and toughness of 100–1000 $Jm^{-2}$ at high water content (90 wt.%) [27]. while toughness reaches 9000 $Jm^{-2}$ with an energy‐dissipative alginate‐${\mathrm{Ca}}^{2+}$ second network [6].

Despite their improved toughness, many strengthened high‐water‐content hydrogels still suffer limitations such as high hysteresis, low reversibility, or slow recovery due to the irreversible failure of sacrificial bonds, and/or the sluggish dynamics of weak secondary networks [21, 28, 29]. Even when non‐covalent bonds [30], nanocomposites [31], or porous frameworks [32, 33] are employed to improve network recoverability, fast recovery times remain elusive due to limitations on the kinetics of network reconstruction via new bond formation [34, 35]. Some elastic gels that rely instead on mechanical mechanisms for energy dissipation such as entangled chains [36, 37, 38] or (un)folding [37] demonstrate impressive recovery and toughness (Table 1). Among them, the so‐called “slide‐ring gels” stand out due to their unique sliding cross‐links which impart exceptional mechanical properties [39, 40, 41, 42]. Slide‐ring gels are derived from polyrotaxanes – mechanically bonded macromolecules comprising a linear polymer guest threaded by multiple macrocycles and stoppered by bulky endgroups to prevent them from de‐threading. When the rings are cross‐linked to form a network, their translational motion with respect to the polymer chain can help reduce inhomogeneities and equalize tension through the network under deformation, leading to high toughness and recoverability, known as the pulley effect [39, 43]. Recently, slide‐ring hydrogels derived from cyclodextrin (CD) rings sparsely threaded on poly(ethylene glycol) (PEG) achieved [44] a notable balance of high toughness and low hysteresis, with a superior tensile strength of 5.5 MPa, high toughness of 2200 $Jm^{-2}$, rapid self‐reinforcement, and nearly 100% reversibility, but at a low water content of 51%. The mechanical performance of these slide‐ring networks significantly declines when allowed to swell to high water content, posing challenges for tissue engineering or load‐bearing applications in extracellular environments or aquatic conditions [45].

A number of slide‐ring hydrogels approach the challenge of maintaining high toughness when swollen through various dual‐crosslinking strategies (Table 1). For example, when CD is functionalized by poly(acrylic acid‐acrylamide), addition of iron(III) salts generates carboxyl‐${\mathrm{Fe}}^{3+}$ coordination bonds, increasing the Young–s modulus to 9.5 kPa and toughness to 10 $kJm^{-2}$ at an equilibrium swelled state with 82.7 wt.% water content, although a large hysteresis with dissipation energy of 7.2 $MJm^{-3}$ and residual strain of 55% with delayed recovery was observed due to the energy‐dissipative dynamic coordination bonds [46]. Dispersion of acrylated polyrotaxanes into poly(ethyl acrylate) ionic liquid increased the ionogel stretchability up to 550% and reduced hysteresis to 7% residual strain [47]. A recent double‐network hydrogel prepared by photo‐crosslinking a polyrotaxane within a Ca^2+^‐alginate network showed excellent tensile stress (199 kPa), elongation (1239%), and toughness (668 $Jm^{-2}$), but also hysteresis and residual strain at higher stretching cycles [48]. A polymerizable pseudorotaxane of acrylated β‐CD and bile acid was photopolymerized with acrylamide to afford tough slide‐ring hydrogels with high stretchability (830%) but low breaking stress (80 kPa) and only 97% recovery in hysteresis cycles even at a low water content of 60 wt.% [49]. Similarly, highly stretchable slide‐ring hydrogels derived from hydroxypropylated α‐CD (HPαCD) grafted with poly(*N*‐isopropylacrylamide‐sodium acrylate) could be elongated to 1400% strain, but displayed only 35 kPa tensile stress even at 80 wt.% water content [50]. A PEG/HPαCD polyrotaxane network chemically crosslinked within an acrylamide‐PEG‐acrylamide network showed high elongation of 2540%, but a low modulus of 35 kPa and high residual strain (19%) [51]. A tough hydrogel with high tensile breaking stress of 7 MPa, 2150% strain at 75wt.% water content has been obtained from photo‐polymerization of acrylamide with an acrylic‐functionalized polypseudorotaxane, but with 5% residual strain after the first cycle [52]. A polyacrylamide network cross‐linked by hydrogen bonds with a carboxymethylated PEG/CD PR showed high stretchability (740%), work of fracture (1100 $kJm^{-3}$), and tensile stress (156 kPa), but at a low water content of 50 wt.% and slow network recovery during cyclic loading‐unloading with 8% residual strain [53]. These examples demonstrate the challenge of maintaining high toughness and good recoverability for slide‐ring hydrogels at high water content.

Recent innovations in highly entangled, sparsely crosslinked networks have yielded hydrogels (Table 1) exhibiting high toughness, stiffness and low hysteresis at high water content (∼90 wt.%) [7, 38, 54, 55]. When chain entanglements greatly outnumber cross‐links, high toughness, fatigue resistance and low hysteresis can emerge even at high water content, enhancing stiffness without embrittling the network due to the transmission of tension during deformation by a slip‐link mechanism [56] analogous to the pulley effect in slide‐ring gels. Inspired by these findings, we hypothesized that the presence of a highly entangled second network in a slide‐ring hydrogel could help mitigate the sliding‐induced swelling that weakens the network in aqueous conditions without over‐compromising on stretchability due to high cross‐link density.

Here we combine two distinct physical slip‐link mechanisms — the pulley effect of a polyrotaxane slide‐ring network and the entangled chains of a sparsely cross‐linked polyacrylamide network — into an interpenetrating double network. These highly entangled slide‐ring double‐network (HESRDN) hydrogels were obtained by photopolymerization of acrylamide and methylene‐bisacrylamide (MBA) swollen in a PEG‐based slide‐ring network with a low inclusion ratio (∼2.8%) of HPαCD rings. The synergy of these two slip‐link mechanisms produces work of fracture (1275 kJm^−3^) and toughness (2020 Jm^−2^) far exceeding the sum of the component networks, as well as near‐complete reversibility (∼99.7%) at >91 wt.% water content. Uniquely, this dual slip‐link architecture confers exceptional resistance to friction‐induced tearing, sustaining continuous friction loading for over 12 h without rupture, an outcome inaccessible to either component network alone or to conventional double networks lacking the combined slip‐link mechanisms. We also present a modeling framework integrating continuum and discrete network descriptions that rationalizes the synergistic toughening in terms of the coupled pulley and sacrificial network mechanisms. These HESRDN hydrogels may be of particular interest in contexts where high water content, robust mechanical performance, and sustained frictional or dynamic loading coincide, such as load‐bearing biomedical implants, underwater soft actuators, and ion‐transport membrane applications.

## 2 Results and Discussion

### 2.1 SRN and HEN Single Networks

**FIGURE 1:**
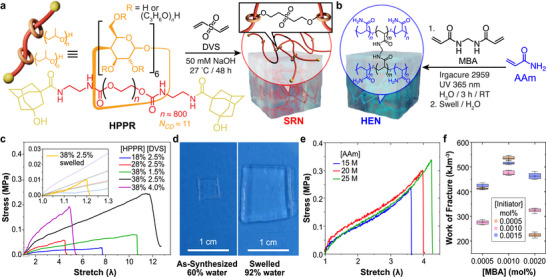
Tough single‐network hydrogels based on ring‐sliding and chain entanglement stress dissipation motifs. (a) Synthesis of a slide‐ring network (SRN) by cross‐linking HPPR with DVS. (b) Synthesis of a highly entangled network (HEN) by photopolymerization of acrylamide (AAm) at high concentration with methylenebisacrylamide (MBA) cross‐linker and Irgacure 2959 photoinitiator at low concentration. (c) Tensile curves for as‐synthesized SRN hydrogels prepared at various concentrations of the HPPR polymer (18‐38 wt.%) and DVS cross‐linker (1.5–4.0 wt.%), compared in the inset with that of the toughest SRN (38% HPPR / 2.5% DVS) after complete swelling in water. (d) Photographs of the optimized SRN (38% HPPR / 2.5% DVS) as‐synthesized and after swelling to 92% water content. (e) Tensile curves of fully swollen HEN hydrogels obtained by photopolymerization of pre‐gel solutions with increasing acrylamide concentration (15–25 M) at a fixed MBA cross‐linker content of 0.001 mol% and a fixed initiator of 0.0005 mol%. (f) Box plots (*n* = 5) of the work of fracture observed for the fully swollen HEN networks synthesized by varying cross‐linker and photoinitiator concentrations between 0.0005–0.002 mol% at a fixed pre‐gel acrylamide concentration of 25M.

We prepared single‐network hydrogels (Figure 1) of the cross‐linked polyrotaxane slide‐ring network (SRN, Figure 1a) and the polyacrylamide‐based highly entangled network (HEN, Figure 1b) to assess their mechanical properties individually and compare them with those of the corresponding interpenetrated double networks.

#### 2.1.1 Synthesis and Mechanical Properties of Polyrotaxane Slide‐Ring Network (SRN)

A series of slide‐ring network (SRN) hydrogels were synthesized (Figure 1a) by cross‐linking HPαCD/PEG polyrotaxane (HPPR) with divinyl sulfone (DVS) in 50 mM NaOH at 27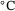 for 48 h. Since the toughest slide‐ring hydrogels based on HPPR have been prepared [44] at high polymer content using sparsely threaded polyrotaxanes, we synthesized the HPPR feed polymer to contain a low inclusion ratio (2.8% of the maximum available binding sites on the polymer backbone) following a previously reported [57] one‐pot synthetic route. The average number of threaded HPαCD rings per chain at this inclusion ratio is approximately 11, determined by ^1^H nuclear magnetic resonance (NMR) spectroscopy (Figure S1).

The as‐synthesized hydrogels exhibit good stretchability and toughness (Figure 1c) at high HPPR content of 18–38% when the DVS cross‐linker concentration in the pre‐gel solution is in the range of 1.5–4.0 wt.%. When [DVS] is fixed at 2.5%, increasing the HPPR content increases the stiffness and ultimate tensile stress of the hydrogels, while the stretchability varies from 8‐fold at 18% HPPR to 5‐fold at 28% HPPR and 12‐fold at 38% HPPR. When [HPPR] is fixed at 38 wt.%, the stiffness also increases as [DVS] rises from 1.5 to 4.0 wt.%, but the 2.5 wt.% sample exhibits the highest work of fracture (1275 kJm^−3^), ultimate tensile strength (UTS, 0.25 MPa), and maximum stretch (λ = 12), since the stiffer sample prepared at 4 wt.% DVS is embrittled, extending to a stretch of only λ = 5. The mechanical properties of our SRN hydrogels are lower than the optimized SR‐0.38 gel of Ito et al. [44], which we attribute primarily to a difference in effective water content; the 38 wt.% polymer content reported for that gel refers only to the PEG backbone and does not include the HPαCD rings and cross‐linker, which together raise the total polymer content to approximately 49 wt.% and lower the water content to approximately 51 wt.%, whereas our SRN hydrogels were prepared at 38 wt.% total HPPR content and correspondingly higher water content of ∼60 wt.%. Nevertheless, these hydrogels exhibit high toughness and remarkable extension ratios, as expected for slide‐ring networks optimized for maximum pulley effect [40]. A summary of the SRN samples obtained with different experimental parameters and the corresponding measurements of mechanical properties, averaged over five trials, is given in Table S1.

A limitation of these single‐network slide‐ring hydrogels with high HPPR content is that they swell considerably in water (Figure 1d) until they are weak and brittle, as shown in the inset of Figure 1c. The optimized SRN (38% HPPR / 2.5% DVS) is 92% water when fully swollen; the modulus shifts down from *E* = 60 kPa to *E* = 0.6 kPa (Figure S2a), stretchability is reduced from 1080% to 10% (Figure S2b), ultimate tensile stress falls from 240 to 8 kPa (Figure S2c), and work of fracture drops from 1550 to 1 ${\mathrm{kJm}}^{-3}$ (Figure S2d). In cyclic loading/unloading tensile tests, the optimized SRN at 60% water content exhibits low hysteresis and high reversibility of 98.7% (Figure S2e). By contrast, our attempts to observe cyclic stretching‐relaxation tensile curves in fully swollen samples failed repeatedly due to fracture. This severe swelling‐induced weakening motivated us to explore the impact of a second interpenetrating highly entangled network.

#### 2.1.2 Synthesis and Mechanical Properties of Polyacrylamide Highly Entangled Network (HEN)

The poly‐acrylamide‐based highly entangled network (HEN) is prepared by photopolymerization of highly concentrated aqueous acrylamide (AAm) with low MBA cross‐linker and Irgacure 2959 photoinitiator content, according to a recent protocol [54] that affords hydrogel networks (Figure 1b) where physical entanglements outnumber covalent crosslinks. The mechanical properties of the HEN hydrogels we obtained were consistent with those reported previously [54]. After complete swelling in water, the HEN gels show good toughness as pre‐gel acrylamide concentration rises from 15 to 20 to 25M (Figure 1e) at fixed MBA cross‐linker and Irgacure 2959 photoinitiator contents of 0.001 and 0.0005 mol%, respectively. At these low cross‐linker and photoinitiator concentrations, the hydrogel samples are stiffer and more extensible at higher starting monomer concentrations (Figure S3a,d,f) due to increasing chain length and entanglement. With acrylamide concentration fixed at 25M, the toughness of the single network was optimized (Figure 1f) by varying the cross‐linker and photoinitiator content between 0.0005–0.002 mol%. As the MBA cross‐linker content rises, the average PAAm chain length and entanglement decrease, leading to stiffer, more brittle samples of lower stretchability and tensile strength (Figure S3b,e,g). The toughest hydrogels were obtained at 0.001 mol% MBA, where mechanical properties were relatively insensitive to photoinitiator content. At this MBA concentration, the toughest samples were those containing only 0.0005 mol% photoinitiator. This optimized HEN hydrogel exhibits low hysteresis, with a high reversibility metric of $U_{2}$/$U_{1}$= 97% in hysteresis loading‐relaxation curves (Figure S3c). All of the samples showed good work of fracture with several exceeding 500 ${\mathrm{kJm}}^{-3}$ (Table S2).

### 2.2 Highly Entangled Slide‐Ring Double Network (HESRDN) Hydrogel

**FIGURE 2:**
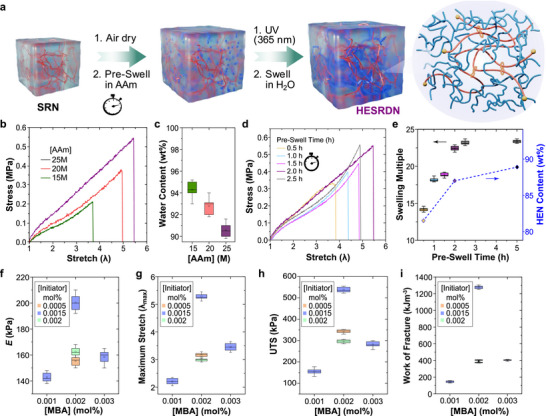
Parameter optimization for a highly entangled slide‐ring double network (HESRDN) hydrogel. (a) Graphical scheme of HESRDN synthesis by UV‐induced photopolymerization of a highly entangled polyacrylamide network within a SRN swollen in AAm and MBA monomers. (b) Tensile curves and (c) box plots (*n* = 5) of HESRDN hydrogels obtained with increasing pre‐cure AAm concentration, prepared from pre‐gels swollen for 2h with photoinitiator and MBA concentrations fixed at 0.0015 and 0.002 mol%, respectively, demonstrate how increasing the pre‐cure AAm concentration increases mechanical strength and decreases water content in HESRDN hydrogels. (d) Tensile curves and (e) box plots (*n* = 5) of swelling multiple demonstrate how mechanical and swelling properties and HEN mass content of the HESRDN hydrogel depend on the pre‐cure swelling time in 25 M AAm, with an optimum pre‐swell time of 2h. Box plots (*n* = 5) illustrate the impact of photoinitiator and cross‐linker concentration on (f) Young's modulus, (g) extensibility, (h) ultimate tensile strength (UTS), and (i) work of fracture of fully swollen HESRDN hydrogels prepared with AAm fixed at 25 M in the pre‐gel soaking solution.

Since both the as‐synthesized SRN and swollen HEN single networks exhibit good stress dissipation as a result of slip‐link systems that can redistribute tension in the network, we were motivated to combine the two networks into an interpenetrating double network (Figure 2) to test the hypothesis that they would exhibit improved toughness in combination when fully swollen at high water content.

#### 2.2.1 Synthesis and Optimization of HESRDN Hydrogel

To synthesize the highly entangled slide‐ring double network (HESRDN) hydrogel, the optimized SRN was air‐dried overnight and then re‐swelled for a period of time in a pre‐gel solution containing AAm, MBA, and Irgacure 2959, then photocured with UV light (365 nm) between two glass slides to produce (Figure 2a) the entangled polyacrylamide interpenetrating second network. All samples were allowed to swell in water at ambient temperature for at least 24 h before undergoing mechanical tests.

We optimized the double network hydrogel properties by preparing a family of HESRDN compositions with variations in (AAm) concentration, cross‐linker concentration, and photoinitiator concentration, as well as swelling time (Table S3). Like the single network HEN, increasing [AAm] in the pre‐gel swelling solution improved both the strength and stretchability of the fully swollen hydrogels (Figure 2b), improving their toughness. The water content in the fully swollen HESRDN gel drops from 94% to 91% as pre‐gel [AAm] rises from 15 to 25M (Figure 2c) at a fixed swelling time of 2 h, indicating the longer, more entangled PAAm chains more effectively mitigate swelling‐induced volume expansion. The optimal 2h pre‐cure swell time was determined (Figure 2d) by comparing the tensile curve profiles of gels that were photocured upon increasing swell times in 0.5h intervals, leading to swelling multiples in the range of 14× to 23× (Figure 2e) relative to the air‐dried SRN. The mass ratio of the secondary network in HESRDN increased with longer pre‐cure swell times, based on gravimetric measurements of the HEN content in HESRDN of 81 wt.% at 0.5h, 86 wt.% at 2h, and 89 wt.% at 5h (Figure 2e). The MBA cross‐linker and Irgacure 2959 photoinitiator concentrations were varied with [AAm] fixed at 25M and the swell time fixed at 2h. Based on the measured stiffness (Figure 2f), stretchability (Figure 2g), tensile strength (Figure 2h), and work of fracture (Figure 2i) of these samples, the optimal pre‐gel cross‐linker and photoinitiator concentrations for the double network were determined to be (MBA) = 0.002 mol% and [initiator] = 0.0015 mol%, respectively, which are relatively higher values than those of the optimized single HEN.

#### 2.2.2 Elasticity of the Optimized HESRDN Hydrogel

**FIGURE 3:**
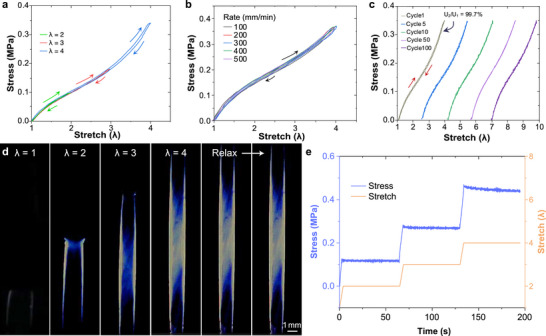
High elasticity of the optimized HESRDN hydrogel. (a) Overlaid tensile curves of HESRDN stretched to extension ratios of λ = 2, 3, and 4 at a strain rate of 100 mm/min. (b) Overlaid tensile curves of HESRDN stretched to an extension ratios of λ = 4 at strain rates of 100–500 mm/min. (c) Tensile curves comparing the 1st, 5th, 10th, 50th and 100th loading‐unloading cycles (λ = 3, 100 mm/min), offset evenly for the sake of visualization. (d) Photographs of HESRDN hydrogel under circular polarizers at different stretch ratios (λ= 2, 3, 4) show birefringence during loading and subsequent relaxation. (e) Plot of stress vs. time during a stress relaxation experiment in which the HESRDN hydrogel is extended to λ = 2, 3 and 4 with 60 s relaxation intervals.

Having optimized the experimental parameters for HESRDN hydrogels, we further explored the elasticity of the toughest samples (Figure 3) based on 38% HPPR / 2.5% DVS SRN pre‐gels swollen for 2h in 25M AAm with 0.002 mol% MBA and 0.0015 mol% photoinitiator.

#### 2.2.3 Hysteresis

Cyclic loading‐unloading experiments at varying extension ratios (Figure 3a), loading/unloading rates (Figure 3b), and over many cycles (Figure 3c) reveal the excellent recoverability of the HESRDN double network hydrogel. Cycling over the range of λ = 1–3, the reversibility in the first loading‐unloading cycle (U_2_/U_1_) is remarkably high at 99.7%, demonstrating minimal hysteresis even compared with other recent outstanding DN hydrogels (see Table S4). Network recovery rates are fast, since this high reversibility is maintained even up to 500 mm/min loading rates (Figure 3b). The hysteresis profiles after 1, 5, 10, 50, and 100 repeated loading‐unloading cycles are offset equally for comparison in Figure 3c, demonstrating consistent mechanical response and minimal accumulation of damage over the course of these measurements.

#### 2.2.4 Stress Relaxation

The high elasticity of the optimized HESRDN is also evident in stress‐relaxation experiments (Figure 3d–e). Photographs of HESRDN hydrogel illuminated through cross polarizers show that the samples become more birefringent under increasing uniaxial tension, indicating an increase in polymer chain alignment, since birefringence and stress are known to be proportionally related to the end‐to‐end distance of the polymer chain orientation [58, 59]. No changes in birefringence are observed during relaxation holds (Figure 3e and Movie S1). Indeed, almost no stress relaxation could be measured (Figure 3e) over 1‐min intervals after loading the hydrogel to extension ratios of 2, 3, or 4, consistent with the high observed elasticity and recoverability of the entangled slide‐ring double network hydrogel.

#### 2.2.5 Performance of the Optimized HESRDN Hydrogel

**FIGURE 4:**
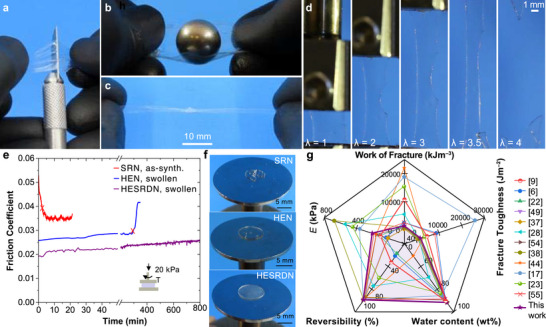
Performance demonstrations of the optimized HESRDN hydrogel. (a) Photograph of a 0.5‐mm thick HESRDN film resisting damage while being stretched over the blade of a knife. (b) Photograph of the same film bearing the load of a 20‐gram steel ball dropped from 15 cm height in a puncture test. (c) Photograph of a hydrogel film withstanding the stresses of knotting and stretching. (d) Photographs of a notched HESRDN hydrogel resisting catastrophic crack propagation beyond a 3× extension ratio. (e) The friction coefficients of SRN, HEN and HESRDN hydrogels at a normal pressure of 20 kPa and angular velocity of 1 rad $s^{-1}$. The SRN and HEN hydrogels ruptured within 2 min and 300 min, respectively. (f) Photographs of HESRDN, SRN, and HEN samples after 900, 2, and 300 min in the friction tear test. (g) Spider plot comparing different tough double‐network hydrogels with built‐in stress storage/dissipation mechanisms along the dimensions of toughness, stiffness, reversibility, water content, and work of fracture [6, 9, 17, 22, 23, 28, 37, 38, 44, 49, 54, 55].

The HESRDN hydrogel samples were further subjected to various stresses to visualize their robust mechanical properties. The tactile feedback of twisting and stretching a thin HESRDN hydrogel resembles that of an elastic rubber band (Movie S2). Thin films (thickness 150 μm) of the HESRDN hydrogel resisted being cut when stretched over the blade of an Exacto knife (Figure 4a), resisted puncture by a 20 g steel ball falling under gravity (Figure 4b and Movie S3), and withstood being tied into an overhand knot and stretched (Figure 4c). A HESRDN hydrogel of 30 mm × 10 mm × 0.5 mm dimensions behaves like a spring when a 100 g mass is suspended on it, vibrating when plucked by hand (Movie S4), exhibiting high toughness and quick recovery. Notably, the mass of polymer supporting this 100 g weight is on the order of only 100 mg. Demonstrating the fracture resistance of the HESRDN hydrogel, a notched sample resists catastrophic crack propagation beyond a 3× extension ratio in a fracture test under uniaxial tension (Figure 4d and Movie S5).

#### 2.2.6 Friction Tear Tests

The optimized single‐ and double‐network hydrogel samples were subjected to continuous shear forces under a 20 kPa normal force in a rheometer at a rate of 1 rad $s^{-1}$, and the friction coefficient was recorded over time (Figure 4e). The friction coefficients of the HESRDN hydrogel were lower than those of either component single network. Lower friction indicates increased thickness of the water‐rich layer between the probe and the hydrogel surface, which is influenced by the density of dangling chains and entanglements in the hydrogel [54]. The SRN and HEN single networks rupture within five minutes and five hours, respectively, whereas the HESRDN hydrogel exhibits continued rupture‐free stability in the friction tear test beyond 12 h (Figure 4e). The photographs in Figure 4f demonstrate the differences in damage sustained by the three hydrogels at the end of the friction tear tests, with a lack of macroscopic visual damage apparent only in the HESRDN hydrogel.

#### 2.2.7 Comparison With Other Tough Hydrogels

The HESRDN hydrogel, with its two interpenetrating networks each offering slip‐link stress dissipation modes, offers a unique combination of toughness, fracture resistance, and reversibility at high water content. The spider plot in Figure 4g compares the HESRDN hydrogel with other outstanding double‐netwrork hydrogels incorporating energy‐dissipation mechanisms along the dimensions of toughness, stiffness, reversibility, water content, and work of fracture [6, 10, 22, 28, 37, 38, 44, 49, 54]. The water content and reversibility of the HESRDN hydrogel are remarkably high while still providing good performance with respect to stiffness, toughness, and fracture resistance. The moderate tensile strength of HESRDN relative to some conventional double network hydrogels with higher UTS (Table 1) reflects the intrinsic softness of the slide‐ring primary network, in which mobile‐ring crosslinks redistribute stress rather than concentrating it; this same architectural feature is responsible for the near‐complete reversibility (99.7%) and exceptional friction tear resistance that distinguish HESRDN from conventional double network hydrogels.

### 2.3 Mechanistic Evaluation of Synergistic Toughening in HESRDN

The work of fracture and toughness of the HESRDN hydrogel are much greater than the sum of the two component single networks SRN and HEN, indicating a synergistic effect of the two networks when they become interpenetrated in a double network. We introduce experimental control networks as a basis for comparison, along with a modeling framework to provide mechanistic insight on the observed synergistic toughening among the two interpenetrating slip‐link networks of the HESRDN hydrogel.

#### 2.3.1 Comparing Slip‐Link Networks With Fixed Cross‐Link Controls

**FIGURE 5:**
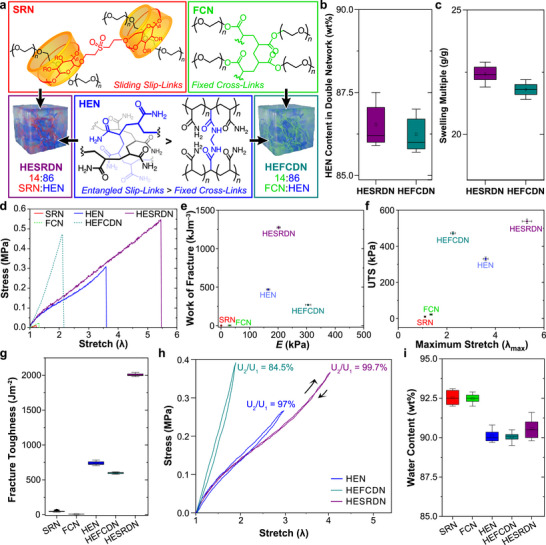
Comparison of component and control networks with the highly‐entangled slide‐ring double network HESRDN. (a) Illustrations of the cross‐links and slip‐links involved in the slide‐ring (SR), highly entangled (HE), and fixed cross‐link (FC) networks. (b) Box plots (*n* = 5) comparing the average percent content of the HEN component observed in the slide‐ring HESRDN and fixed‐crosslink HEFCDN hydrogels, expressed as a wt.% of total polymer content. (c) Box plots (*n* = 5) comparing the swelling multiples of the HESRDN and HEFCDN control hydrogels. (d) Overlaid tensile curves of optimized SRN, FCN, HEN, HEFCDN, and HESRDN hydrogels. (e) Scatter plot of work of fracture vs. Young's modulus (*E*) for SRN, FCN, HEN, HEFCDN, and HESRDN samples. (f) Scatter plot of ultimate tensile strength vs. maximum stretch for SRN, FCN, HEN, HEFCDN, and HESRDN hydrogels. Error bars in the scatter plots represent the standard deviation of the mean (*n* = 5). (g) Box plots (*n* = 5) of notched fracture toughness measurements obtained for the fully swollen SRN, FCN, HEN, HEFCDN, and HESRDN hydrogels. (h) Overlaid cyclic tensile curves comparing the hysteresis profile of HEN, HEFCDN, and HESRDN hydrogels, each elongated to approximately 80% maximum stretch at a rate of 100 mm/min. (i) Box plots (*n* = 5) of water content measurements for the fully swollen SRN, FCN, HEN, HEFCDN, and HESRDN hydrogels.

To support a mechanistic understanding of the double‐slip‐link network HESRDN, we prepared several control hydrogels with slip links disabled in the primary and secondary networks (Table S5). A fixed cross‐link network (FCN) derived from PEG‐diacrylate, equal to SRN in water content, was employed to synthesize the corresponding highly entangled fixed cross‐link double network (HEFCDN, Figure 5a) by the same drying‐swelling‐photocuring process used for HESRDN, yielding a hydrogel of 14:86 host:guest mass ratio (Figure 5b) and 22 g/g swelling multiple (Figure 5c), closely matching those of HESRDN. From the tensile curves (Figure 5d), the observed values of HESRDN hydrogel stretchability (λ_max_ = 5.3±0.2), ultimate tensile strength (538±12), stiffness (*E* = 202±6 kPa), and toughness (1275±18 kJm^−3^) all exceed the sum of their corresponding component single networks (Figure S4). Scatter plots of toughness vs. Young's modulus (Figure 5e) and ultimate tensile strength vs. extensibility (Figure 5f) further visualize these synergistic improvements of the slide‐ring double network. The fracture toughness of the HESRDN hydrogel observed in notched tension tests is especially enhanced compared to the SRN and HEN single networks (Figure 5g), exceeding 2000 Jm^−2^ compared to 52 and 745 Jm^−2^, respectively.

By contrast, the fixed‐crosslink control, HEFCDN, is stiffer (*E* = 300 kPa) with lower fracture toughness (Figure 5e,g) and extensibility (Figure 5e) than even the single‐network HEN (Table S5). Unlike the enhancement in recoverability observed in the case of HESRDN ($U_{2}/U_{1}$=99.7%), the fixed‐crosslink host network degrades the recoverability of HEFCDN ($U_{2}/U_{1}$=84.5%) relative to that of the HEN single network ($U_{2}/U_{1}$=97%), evident in the overlaid cyclic loading‐unloading tensile curves in Figure 5h. Given their identical HEN content (Figure 5b) and water content (Figure 5i), the stark differences in mechanical performance of HEFCDN and HESRDN implicates the properties of slide‐ring host network in the observed synergistic toughening of HESRDN. Unlike conventional double network hydrogels in which the primary network serves as a sacrificial network that dissipates energy through irreversible bond rupture (leading to permanent damage and hysteresis), the slide‐ring primary network of HESRDN fulfills the same sacrificial role without sustaining irreversible damage, since mobile‐ring crosslinks can slide along the polyrotaxane backbone to accommodate local stress concentrations rather than rupturing; a unique feature that likely accounts for the near‐complete reversibility (99.7%) that distinguishes HESRDN from conventional double network hydrogels. These comparisons demonstrate that the slide‐ring mechanism works effectively in the HESRDN design: the mobile‐ring crosslinks enable reversible sacrificial network behavior, conferring synergistic toughening and near‐complete recoverability that are inaccessible to the corresponding fixed‐crosslink double network control.

As an additional control lacking slip‐links in the secondary network, a low entangled slide‐ring double network (LESRDN) was prepared according to the same protocol, except that the content of MBA cross‐linker in the PAAm‐based secondary low entangled network (LEN) was increased to 1.0 mol% to ensure that fixed cross‐links would outnumber entangled slip‐links (Figure S5). Although the 13:87 mass ratio of primary:secondary networks is similar to that of HEFCDN and HESRDN (14:86), the water content of LEN (66 wt.%) and LESRDN (70 wt.%) are much lower, since their higher cross‐link densities limit swelling. Thus, the LESRDN hydrogel is expectedly stiffer (*E* = 925 ± 15 kPa) with comparable ultimate tensile stress of 430 ± 10 kPa, but it is much more brittle than the highly entangled counterparts, with relatively low stretchability of 1.35 ± 0.5), work of fracture (78 ± 6 ${\mathrm{kJm}}^{-3}$), and fracture toughness (182 ± 12 ${\mathrm{Jm}}^{-2}$) despite having much higher polymer content. Photographs of notched tests from each of the control samples are presented in Figure S6. We note that the lower water content of LESRDN (70 wt.%) relative to HESRDN (91 wt.%) is a thermodynamically inseparable consequence of its higher crosslink density, since increased crosslinking necessarily restricts equilibrium swelling; this variable mismatch prevents a perfectly controlled single‐variable comparison, and the brittleness of LESRDN likely reflects the combined effects of reduced entangled slip‐links, increased fixed crosslink density, and lower water plasticization. Despite this caveat, the poor mechanical performance of LESRDN relative to HESRDN supports the conclusion that entangled slip‐links in the secondary network are important contributors to high stretchability and toughness, consistent with the stronger evidence provided by the better‐matched HEFCDN control.

#### 2.3.2 Modeling Framework for Slide‐Ring Double Networks

**FIGURE 6:**
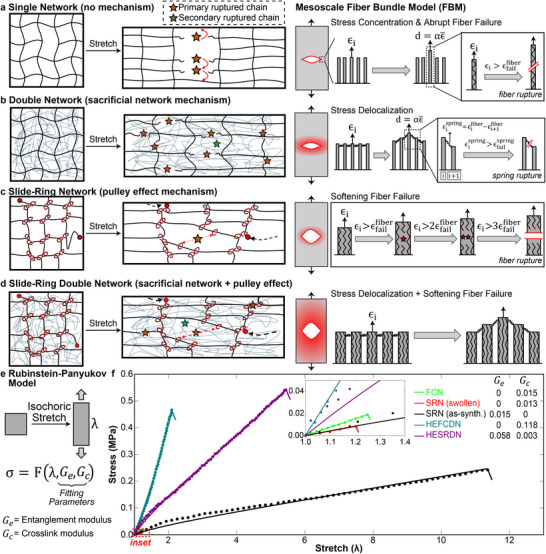
Schematic and modeling framework illustrating distinct failure mechanisms in polymer networks captured with a discrete mesoscale fiber bundle model (FBM) and continuum‐scale Rubinstein–Panyukov model. (a) A single network with covalent cross‐links exhibits localized chain rupture and brittle failure, capture by abrupt fiber rupture in the FBM. (b) A covalent double network is toughened by a sacrificial, stretchable secondary network that delocalizes damage, captured in the FBM by inter‐fiber springs that transfer local stresses to adjacent fibers. (c) A slide‐ring network is toughened by the pulley effect, where the translation of mobile cross‐links redistributes stress to enhance extensibility and delay the onset of failure at ring junctions. Damage is captured in the FBM by a softening fiber failure mechanism involving multiple steps of local damage and softening before complete fiber rupture. (d) A slide‐ring double network integrates both stress redistribution and energy dissipation mechanisms, resulting in exceptional toughness and stretchability, captured in the FBM by combining the inter‐fiber springs and softening fiber failure elements. (e) Summary of parameters in the continuum‐scale Rubinstein–Panyukov model. (f) Scatter plots comparing experimental stress–stretch curves and fits to the Rubinstein–Panyukov model, with failure points mapped onto the curves using the FBM.

To provide mechanistic insights into the enhanced toughness and extensibility of the HESRDN hydrogel, we developed a modeling framework that integrates both continuum and discrete approaches. The goal was to rationalize the observed differences in failure behavior between various network designs and to identify how specific architectural features, such as slide‐ring mobility or sacrificial bonding, contribute to the macroscopic mechanical properties. Figure 6 illustrates the evolution of network architecture and stress response under uniaxial stretching for four distinct types of hydrogel networks (single network, slide‐ring network, double network, slide‐ring double network), how their mechanisms of microstructural damage are captured by a fiber bundle model [60, 61] (FBM), and fits of the experimental data representing each case to a continuum‐scale Rubinstein–Panyukov [62] constitutive framework. Details of the modeling framework are provided in Figures S7– S11 and associated discussion of the Supporting Information.

Figure 6a illustrates the microstructure of a conventional single‐network hydrogel with fixed covalent cross‐links under uniaxial stretch, where chain rupture is highly localized and brittle failure occurs rapidly due to the absence of stress redistribution pathways. Figure 6b illustrates how a covalent double network hydrogel with a soft, stretchable and highly entangled secondary network dissipates energy and delays failure of the whole network by delocalizing stress through sacrificial chain scission in the the swollen primary network before the damage initiation in secondary network [9, 10, 27]. This happens while the secondary network can also dissipate and delocalize stress through reptation. The FBM is a one‐dimensional discrete framework consisting of linear elastic fibers (stiffness E) connected by shear springs (stiffness k), with rupture governed by their respective failure strains. The dimensionless parameter $\theta =\varepsilon_{\text{fail}}^{\text{spring}}/\varepsilon_{\text{fail}}^{\text{fiber}}$ is introduced to characterize the relative extensibility of the secondary network. In the FBM, brittle single networks (Figure 6a) are modeled with k=0, while highly stretchable covalent double networks (Figure 6b) correspond to soft, extensible springs with k<E and θ≫1.

Figure 6c illustrates how the pulley effect toughens a slide‐ring single network by allowing the cross‐linked rings to redistribute tension along the polymer backbone, significantly enhancing extensibility before damage onset [63]. Uniquely, slide‐ring networks can adapt to damage at a mobile cross‐link by the translational motion of adjacent mobile cross‐links [64], leading to local softening rather than complete failure at the damage site [65]. To account for this characteristic ductility of a slide‐ring network, we incorporate a multistage softening mechanism into the FBM, which mimics the gradual softening by reducing fiber stiffness in progressive steps before complete rupture. Finally, Figure 6d illustrates how the sacrificial network and pulley effect mechanisms combine in a slide‐ring double network, achieving exceptional toughness and extensibility through coupled mechanisms. The mobility of sliding cross‐links allows stress to be redistributed more evenly across the host network, preventing stress concentration at individual chains, and reducing primary network's chain rupture, while the secondary network constrains the elasticity and re‐distributes stress. This dual mechanism reduces the likelihood of crack initiation and propagation, allowing the material to withstand large deformations. As a result, the combination of adaptive cross‐link mobility and energy dissipation provides an effective strategy for achieving extremely high stretchability without sacrificing structural integrity.

While both slide‐ring (SRN) and highly entangled (HEN) hydrogels generally behave elastically at small stretch, their tensile responses differ notably at larger deformations. HEN gels exhibit a clear nonlinear strain‐stiffening trend, which we attribute to the limited ability of the network to rearrange before chains reach their full extension and damage mechanisms are activated. In contrast, SRN gels do not exhibit pronounced stiffening. As shown in previous models [66], this behavior arises from the sliding of mobile cross‐links, which allows chains to redistribute tension and delay the onset of chain stretching and stiffening. To capture these differences within a continuum description, we incorporate the Rubinstein–Panyukov constitutive framework [62], which explicitly separates elastic contributions arising from permanent crosslinked junctions (quantified by the modulus $G_{c}$) from topological or entangled junctions (quantified by the modulus $G_{e}$). This separation enables a more mechanistically informative interpretation of the elastic response of complex hydrogel networks, particularly those involving entanglements or sliding junctions. By fitting the Rubinstein–Panyukov model to the experimental stress–stretch data (Figure 6e,f; derivation in the Supporting Information), we identify the dominant elastic mechanisms governing each hydrogel system. The fully crosslinked network (FCN) and the swollen slide‐ring network (swollen SRN) both exhibit elastic responses dominated by crosslinked junctions, with negligible contribution from entanglements ($G_{e}\approx 0$). While entangled ring polymers are present in the swollen SRN, swelling and bulky chain‐end stoppers suppress effective reptation, rendering the elastic response effectively crosslink‐like. In contrast, the as‐synthesized (unswollen) slide‐ring network exhibits an entanglement‐dominated elastic response, with the fitted crosslink modulus $G_{c}$ being essentially zero (Figure S11). The ability of sliding junctions to redistribute stress via reptation enables large extensibility and suppresses strain stiffening, explaining the enhanced stretchability of the unswollen SRN relative to its swollen counterpart.

Applying the Rubinstein–Panyukov framework to the double‐network hydrogels further highlights the role of architecture. The conventional covalent double network (HEFCDN) remains dominated by crosslinked elasticity ($G_{e}\approx 0$), indicating that elastic energy storage up to rupture is primarily carried by the densely crosslinked primary network. In contrast, the slide‐ring double network (HESRDN) exhibits an entanglement‐dominated response, with $G_{e}$ nearly an order of magnitude larger than $G_{c}$. This shift reflects the synergistic interaction of two sliding networks, which promotes stress redistribution and maximizes elastic energy storage prior to failure, consistent with the exceptional extensibility and toughness observed experimentally. The specific failure points on each stress–stretch curve are input from the FBM, thereby converging the continuum and discrete descriptions on the experimental data. The good agreement between the Rubinstein–Panyukov fits, the FBM predictions, and the experiments supports the interpretation of exceptional toughness in HESRDN as arising from the synergistic combination of sliding‐enabled entanglement elasticity and stress delocalization associated with the sacrificial network architecture.

## 3 Conclusions

We have developed a highly entangled slide‐ring double network (HESRDN) hydrogel that is synergistically toughened by a combination of mobile “slip‐link” cross‐links in the component polyrotaxane and polyacrylamide networks. Under tension, the sliding of these mobile cross‐linking points redistributes stress as the polymer segments become more oriented, leading to entropy loss with almost no energy dissipation during mechanical deformation. This double slip‐link mechanism leads to high reversibility with fast recovery times when stress is relieved from the double network, as well as high work of fracture and toughness. The double slip‐link network is substantially more tough, fracture resistant, and recoverable than control double networks lacking either slide‐ring slip‐links in the primary network or highly entangled slip‐links in the secondary network. Based on continuum and discrete models of the system, we attribute the excellent stretchability, reversibility, toughness, and fracture resistance of HESRDN to the synergistic combination of the pulley/slip‐link effects that toughen both the primary and secondary networks, together with the stress delocalization / sacrificial bond mechanisms characteristic of most double networks. Thus, the strategic association of the slide‐ring and highly entangled networks delivers a hydrogel material with the promising features of high water content with excellent toughness and reversibility.

Several directions for future work emerge from this study. Systematic variation of the HPαCD inclusion ratio, HPPR chain length, and CD content in the polyrotaxane precursor represent promising directions for future work aimed at further tuning the mechanical properties of HESRDN hydrogels and expanding the accessible range of slip‐link density and network architecture. The mechanical stability of HESRDN across a range of pH and ionic strength conditions, which is expected to be relatively robust given the absence of ionizable groups in the neutral PAAm secondary network, warrants systematic investigation in future work targeting specific application environments. In the context of future applications, this double slip‐link strategy may be adapted for other polymer chemistries and help reduce costs in advanced hydrogel materials by reducing the polymer content required to maintain sufficiently high mechanical performance. The combination of high water content with high toughness, fracture resistance, and reversibility may be of particular interest in application domains where cost savings, sustainability, aqueous load‐bearing or biomedical interventions, or water‐ and ion‐transport processes for clean energy are needed.

## 4 Experimental

### 4.1 Materials

Polyethylene glycol (PEG, MW 35 kDa, 818892), 2‐(Hydroxypropyl)‐alpha‐cyclodextrin (HPαCD, 0.6MS, 390690), Acrylamide (AAm, A8887), 2‐Hydroxy‐4‐(2‐hydroxyethoxy)‐2‐methyl‐propiophenone (Irgacur 2959, 410896), N,N'‐methylenebisacrylamide (MBA, M7279), poly(ethylene glycol) diacrylate (PEGDA, Mn 700, 455008) and dialysis membrane (MWCO 14 kDa, D9777) were purchased from Sigma–Aldrich. 3‐Hydroxy‐1‐adamantane carboxylic acid (AdCOOH, H1255), divinyl sulfone (DVS, D0959) and anhydrous ethylenediamine (E0037), were purchased from TCI America. 4‐(4,6‐dimethoxy‐1,3,5‐triazin‐2‐yl)‐4‐methylmorpholinium chloride (DMTMM, 99.1%) was purchased from Chem Impex International, Inc. *N*,*N*'‐Carbonyldiimidazole (CDI) was purchased from Oakwood Chemicals. Tetrahydrofuran (THF), and sodium hydroxide (NaOH) were purchased from Fisher Scientific. Ethanol was purchased from Decon Laboratories, Inc. All materials were used as‐received without further purification. Water was collected from a MilliQ water purifier and degassed with purging nitrogen prior to use. Circularly polarized filters, acrylic sheets, silicone rubber sheets and Teflon sheets were purchased from Amazon. Amine‐terminated PEG (PEG(NH_2_)_2_) was synthesized according to a reported procedure [67]. HPPR was synthesized according to a reported one‐pot protocol [57] from PEG(NH_2_)_2_ (1.65 g) and HPαCD (1.416 g) in phosphate‐buffered saline (PBS, 20 mL), incubated at 4 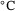 for 2.5 d and then stoppered with AdCOOH (30 mg) and DMTMM (132 mg, added in two equal portions at ambient temperature over 2 d). HPPR was isolated after dialysis in water for 2 d and lyophilization.

### 4.2 Instrumentation

^1^H NMR spectra were recorded on a Bruker Avance 300 MHz nuclear magnetic resonance spectrometer in (CD_3_)_2_SO at ambient temperature. Mechanical properties were measured using a dynamic mechanical analyzer (MCR‐702, Anton Paar GmbH). Freeze‐dried samples were prepared in a Labconco 4.5 freeZone lyophilizer (Labconco corporation). Digital images and movies were captured by Canon EOS 6D camera.

### 4.3 Synthesis of Hydrogels

#### 4.3.1 Synthesis of SRN Hydrogels

A typical SRN hydrogel (with [HPPR] 38 wt.% and [DVS] 2.5 wt.%) was synthesized by dissolving HPPR polymer (0.15 g) into 0.05 M NaOH solution (0.25 mL), followed by the addition of divinyl sulfone (DVS) (10 mg) into the resulting mixture. The final mixture was transferred into a mold made of 2 Teflon‐coated glass slides and silicon rubber spacer tightly fixed by binder clips. The mold was kept at 27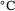 under humidity conditions (relative humidity 88±2%) for 2 days to allow gelation. Other SRN gel formulations were synthesized accordingly at varying HPPR polymer (18–38 wt.%) and DVS crosslinker (1.5–4 wt.%) concentrations. Molds containing SRN hydrogels were stored in a sealed box until further characterization.

#### 4.3.2 Synthesis of HEN Hydrogel

The HEN hydrogel was synthesized by UV initiated photopolymerization reaction of a pre‐gel solution. The pre‐gel solution consists AAm monomer, MBA crosslinker and Irgacur 2959 initiator and water in a certain ratio (Table S2). For a typical formulation, 0.45 g AAm was dissolved in 0.25 mL water (25M solution) by vigorous mixing at 27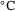. Separately 0.1M of MBA crosslinker solution and 0.1 M of Irgacur 2959 initiator solution were freshly prepared with water and ethanol respectively. To the AAm solution, MBA (1.3 μL, 2.0 x 10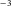 mol% with respect to AAm) and Irgacur (0.9 μL, 1.5 × 10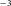 mol% with respect to AAm) solutions were added and vortexed for 30 s. The solution was then purged with nitrogen and then sonicated at 30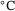 for 3 min. Then the pre‐gel solution was injected into a glass mold with a silicone rubber spacer, tightly fixed by binder clips and kept under UV irradiation (365 nm, 5 mW ${\mathrm{cm}}^{-2}$) for 3 h. Finally, the hydrogel was peeled off from the mould and immersed into excess water for 24 h to swell to equilibrium. The equilibrium swollen sample was kept in a sealed vial for subsequent testing. Similarly, different HEN hydrogel samples were prepared by varying AAm (15–25 M), MBA (0.5–3.0 × 10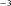 mol%) and Irgacur 2959 initiator (0.5–2.0 × 10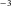 mol%) concentrations in the pre‐gel solution.

#### 4.3.3 Synthesis of HESRDN Hydrogel

To synthesise HESRDN, a pre‐dried SRN was soaked into the pre‐gel solution used to synthesise HEN and then photo‐cured with UV light. For a typical formulation, a pre‐gel soaking solution was prepared by mixing 0.45 g AAm in 0.25 mL of water (25 M), MBA (1.3 μL, 2.0 × 10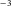 mol% with respect to AAm) and Irgacur 2959 (0.9 μL, 1.5 × 10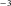 mol% with respect to AAm) followed by purging with nitrogen gas and sonication at 30 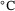 for 3 min. Subsequently, a pre‐dried piece of HPPR‐SN gel was immersed into the pre‐gel solution and the container was kept in dark for a period of time (typically 2h, Table S3) at 27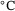 for swelling. Afterward, the swelled HPPR‐SN gel was placed between two glass slides with a silicone rubber spacer, tightly fixed by binder clips and kept under UV irradiation (365 nm, 5 mW ${\mathrm{cm}}^{-2}$) for 3 h to form PAAm network into the HPPR network. Finally, the hydrogel was peeled off from the mold and immersed into water for 24 h to swell to equilibrium which result in the HESRDN hydrogel. The equilibrium swollen sample was kept in a sealed vial for further testing. Similarly different HESRDN hydrogels were synthesized by varying AAM, MBA, Irgacur 2959 initiator concentrations in the pre‐gel solutions and also varying the swelling time of the dry SRN into the pre‐gel soaking solutions.

#### 4.3.4 Synthesis of HEFCDN Control Hydrogel

Fixed crosslinked network (FCN) hydrogel was synthesized by phtocrosslinking a poly(ethyleneglycol) diacrylate pre‐gel solution. 8 wt.% of PEGDA was dissolved in water and purged with nitrogen gas followed by mixing of 0.005 wt.% of Irgacure 2959 initiator. Then the pre‐gel solution was injected into a glass mold with a silicone rubber spacer, tightly fixed by binder clips and kept under UV irradiation (365 nm, 5 mW ${\mathrm{cm}}^{-2}$) for 3 h to obtain FCN single network hydrogel. Subsequently a pre‐dried piece of FCN gel was soaked into the AAm pre‐gel solution used to synthesise HESRDN above and then photo‐cured with UV light followed by swelling in water to obtain the HEFCDN control hydrogel.

#### 4.3.5 Synthesis of LESRDN Control Hydrogel

LESRDN control hydrogel was synthesized following the same protocol used for HESRDN, with only change in the [MBA] of 0.1 mol% with respect to AAm in the pre‐gel soaking solution.

### 4.4 Mechanical Testing

For tensile test, a rectangular sample (10 × 3 × 0.35 ${\mathrm{mm}}^{3}$) was mounted to the solid rectangular fixtures (SRF) of a dynamic mechanical analyzer (MCR 702). The sample was uni‐axially stretched at a speed of 100 mm $\min^{-1}$ until failure. Stress value was obtained by dividing force by initial cross‐sectional area of the sample. Strain was calculated by dividing elongation by initial length of the sample and reported as stretch ($\lambda =L/L_{0}$), a dimensionless measure of elongation. The stiffness (E) was measured from the initial slope of the stress–stretch curve. The work of fracture value was calculated from the area under the tensile curve using Origin Pro software. To calculate toughness (Γ), a tensile data was recorded for a sample with a notch. Then the area under the curve (U) was calculated until critical strain from where the crack grows catastrophically and finally Γ was calculated by Γ = H × U; where H is the initial sample length. For cyclic loading and unloading curves or hysteresis loop, a rectangular sample (10 × 3 × 0.35 ${\mathrm{mm}}^{3}$) was mounted to the SRF and tensile loading‐ unloading cycles were performed up to a certain stretch with a test speed of 100 mm $\min^{-1}$. Similarly, the hysteresis cycles were also recorded with different extension rates of 100–500 mm $\min^{-1}$. For stress relaxation experiments, a rectangular hydrogel sheet was stretched up to a pre‐defined strain and the force exerted was recorded for a 1 min relaxation period. All of the tensile data, hysteresis cycles and stress‐relaxation data was recorded in a moisture‐saturated environment within a custom‐made humidity chamber to mitigate effects of drying. The friction coefficient was measured by a MCR 702 rheometer equipped with a parallel plate attachment. Hydrogel samples were cut into a circular disks of diameter 8 mm and glued to the bottom stage of 25 mm diameter and submerged in water. The diameter (*D*) of the top plate was 8 mm. Subsequently, torque (*T*) was measured under shear rate of 1 rad $s^{-1}$ at an applied axial force (*P*) of 1 N (20 kPa). The friction coefficient was then calculated by *8T/3DP* [54].

### 4.5 Swelling Measurements

Swelling multiple and water content of the hydrogel samples were measured by gravimetric estimation. The air‐dried HPPR‐SN hydrogel weighing $w_{1}$ was soaked in the AAm pre‐gel soaking solution for a certain time; and the weight of the swollen hydrogel become $w_{2}$. The swelling multiple in weight S was calculated by the following formula:

$$S=w_{2}/w_{1}$$

Similarly, water content (W) of the hydrogel was calculated by the following equation:

$$W=\left(m_{s}-m_{d}\right)/m_{s}\times 100\%$$

where, $m_{s}$ is the weight of equilibrium swollen hydrogel and $m_{d}$ is mass of the hydrogel after complete drying.

### 4.6 Birefringence Imaging

The birefringence images of sample during loading and relaxation were recorded by a home‐made circular polarizing optical setup. Two circular polarizer films were positioned at both the front and rear of the sample keeping them between a white light source and a camera. A video was recorded during stretching of a HESRDN hydrogel sample to 4 times of its initial length (λ = 4) and subsequent relaxation step using the polarizer setup.

### 4.7 Statistical Analysis

All measurements were performed on five independent samples (n=5) without preprocessing, transformation, or normalization of the raw data. No outliers were excluded from the analysis. Data are presented as mean ± standard deviation (SD), with the standard deviations of the mean for measured values reported in Tables S1– S3. The statistical spread of measurements is visualized using box plots, where the box represents the interquartile range, the center line represents the median, and the whiskers represent the full range of observations. No formal hypothesis testing or tests for statistically significant differences between groups were performed, as the mechanical differences between the hydrogel compositions studied are large relative to the measurement variability, as evident from the box plots and standard deviations reported. All statistical analysis and visualization were performed using OriginPro software (OriginLab Corporation, Northampton, MA, USA).

## Conflicts of Interest

The authors declare no conflicts of interest.

## Supporting information


**Supporting File 1**: advs75525‐sup‐0001‐SuppMat.pdf.

**Supporting File 2**: advs75525‐sup‐0002‐MovieS1.mp4.

**Supporting File 3**: advs75525‐sup‐0003‐MovieS2.mp4.

**Supporting File 4**: advs75525‐sup‐0004‐MovieS3.mp4.

**Supporting File 5**: advs75525‐sup‐0005‐MovieS4.mp4.

**Supporting File 6**: advs75525‐sup‐0006‐MovieS5.mp4.

## Data Availability

The data that support the findings of this study are available from the corresponding author upon reasonable request.
