# Supplementary material for: Synergistic Dual Slip‐Link Toughening of a Water‐Rich Double Network Hydrogel Combining Slide‐Ring and Highly Entangled Networks
Source: Adv Sci (Weinh). 2026 May 5;13(42):e75525. doi: 10.1002/advs.75525 (PMC13335573; doi:10.1002/advs.75525)
Supplement: Supplementary file 1 — Supporting File 1: advs75525‐sup‐0001‐SuppMat.pdf. [file ADVS-13-e75525-s006.pdf]

Supporting Information

Synergistic Dual Slip-Link Toughening of a Water-Rich Double Network Hydrogel Combining Slide-Ring and Highly Entangled Networks

Subhankar Mandal[1], Saleh Assadi[2], Aseem Milind Visal[3], Ignacio Lorente Montero[1], Franck J. Vernerey[*2], and Carson J. Bruns[*1,2]

[1]ATLAS Institute, University of Colorado Boulder
[2]Department of Mechanical Engineering, University of Colorado Boulder
[3]Materials Science and Engineering Program, University of Colorado Boulder
[*]franck.vernerey@colorado.edu; carson.bruns@colorado.edu

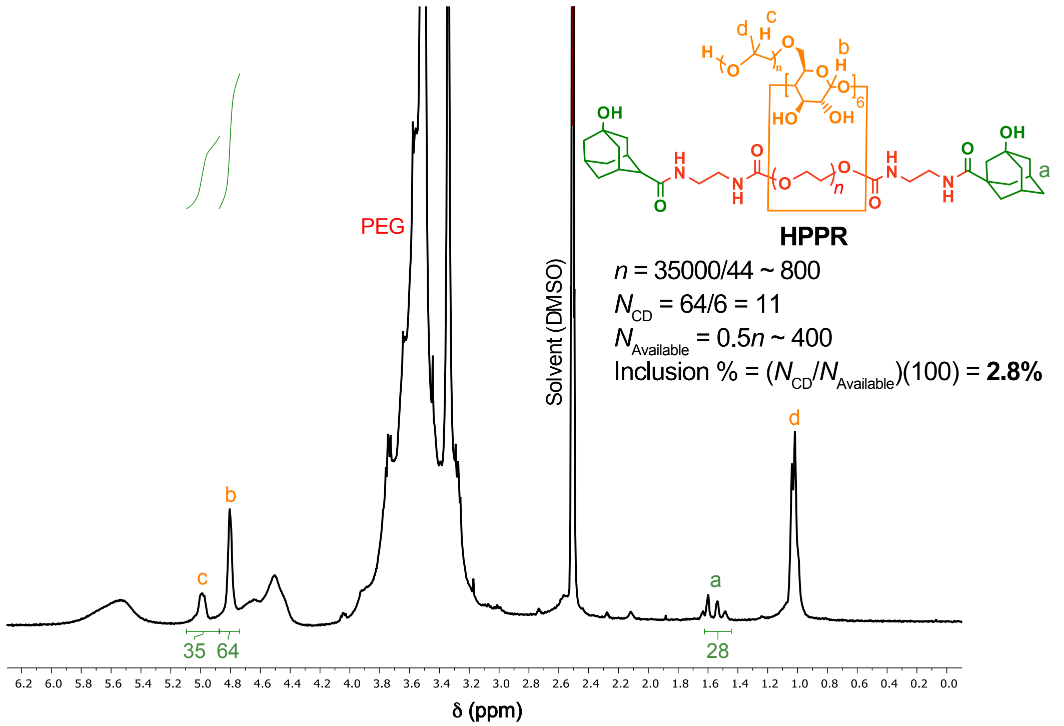

Figure S1: ^{1}H NMR of HPPR in DMSO-d6. The number of threaded CD rings is calculated by comparing the integrated area of the signal attributable to the 28 C-H protons of the adamantyl stopper (signal a) with that of the six anomeric C1 protons of the CD ring (signal b), giving an average total of ∼11 CD rings per chain. Since two repeating units of PEG occupy the CD cavity, the total number of available binding sites is $N_{Available} = 0.5n = 400$ and the inclusion ratio was calculated by $(N_{CD}/N_{Available}) \times 100\% = 2.8\%$.

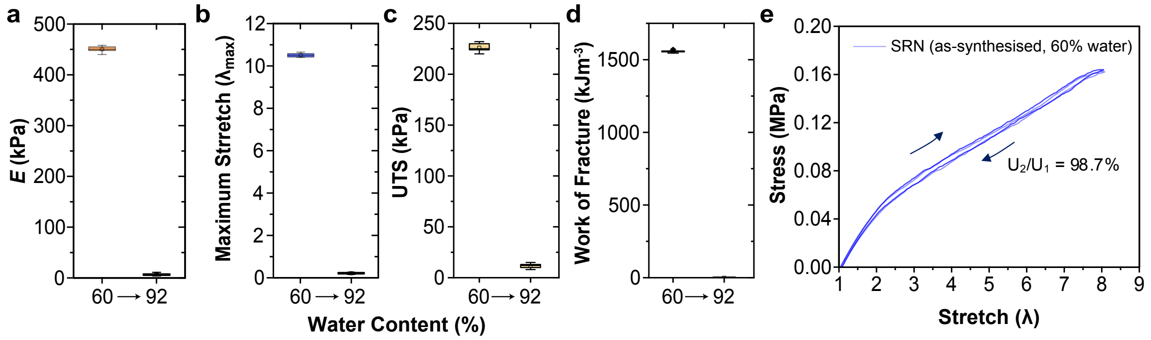

Figure S2: Mechanical properties of the optimized SRN hydrogel prepared from 38 wt% HPPR and 2.5 wt% DVS. The as-synthesized (60% water content) and fully swollen (92% water content) samples are compared with respect to a) stiffness, b) maximum stretch, c) ultimate tensile stress (UTS), and d) work of fracture. e) Three consecutive overlaid stretch-relaxation stress-strain curves demonstrating the low hysteresis and reversibility of 98.7% for the as-synthesized network at a rate of 100 mm/min.

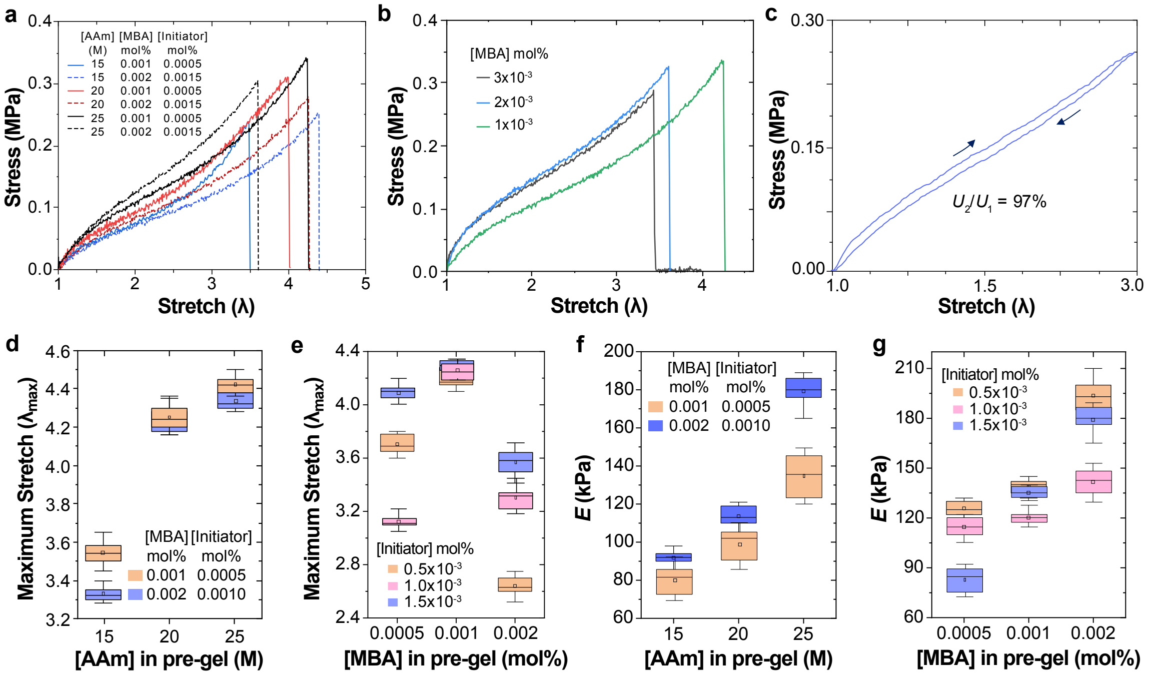

Figure S3: Mechanical properties of highly entangled PAAm hydrogels. a) Representative tensile curves for hydrogels with variations in AAm monomer, MBA cross-linker, and photo-initiator concentrations. b) Representative tensile curves for hydrogels with variations in [MBA] concentration from 0.001 to 0.003 mol%. c) Tensile loading- unloading cycles of the optimized PAAm-SN hydrogel up to $\lambda = 3$, U_2/U_1 denoting the reversibility during the hysteresis cycles. d) Box plots of maximum stretch at three different AAm concentration and two different [MBA]/[Initiator] concentrations. e) Box plots of maximum stretch at three different MBA concentrations and three different photoinitiator concentrations. f) Box plots of hydrogel stiffness at three different AAm concentration and two different [MBA]/[Initiator] concentrations. g) Box plots of hydrogel stiffness at three different MBA concentrations and three different photoinitiator concentrations.

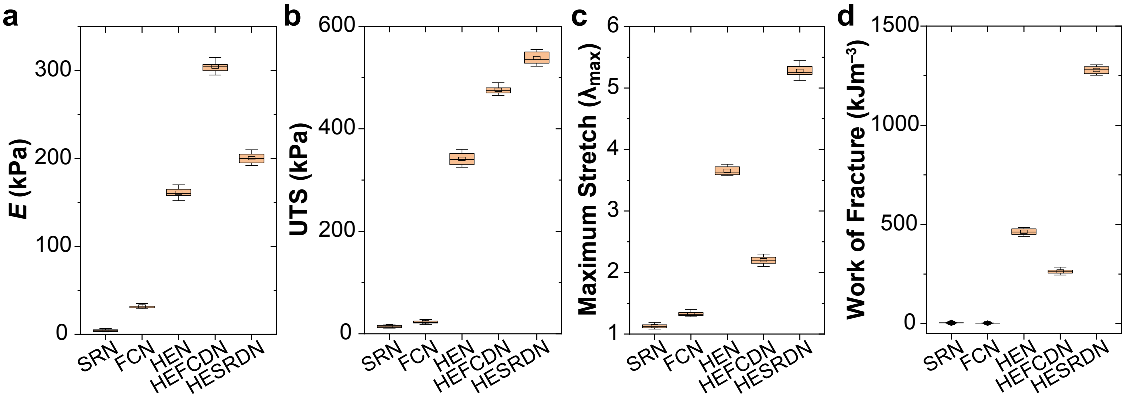

Figure S4: Comparison of the mechanical properties measured for the optimized single and double network hydrogels with regard to a) elastic modulus, b) ultimate tensile stress (UTS), c) maximum stretch, and d) work of fracture.

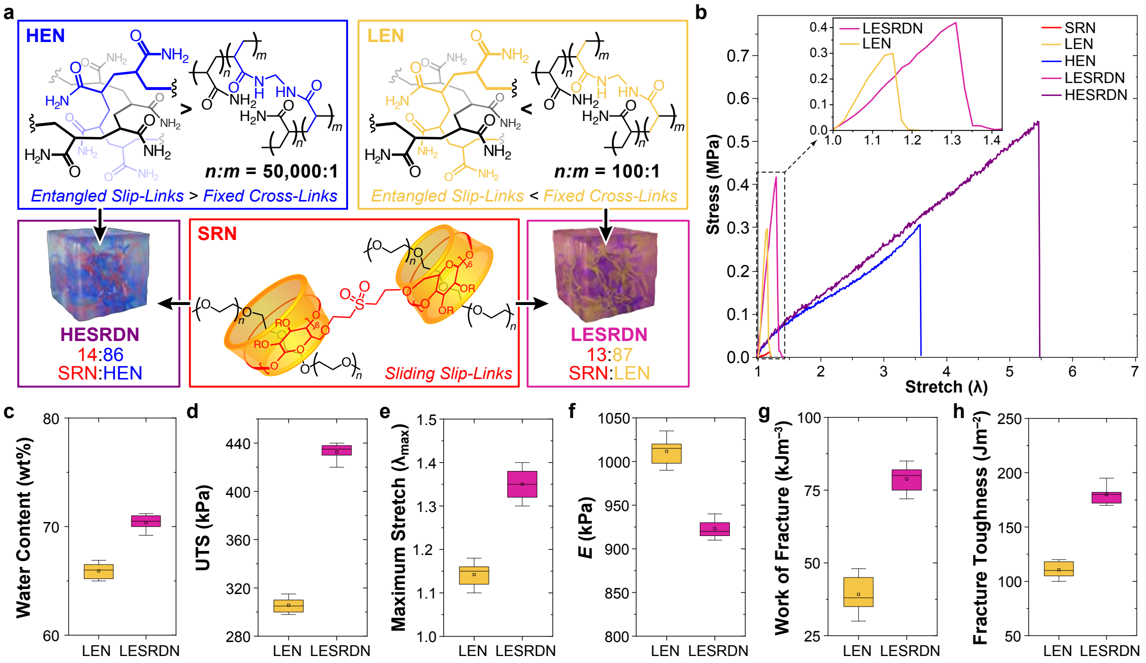

Figure S5: Comparison of mechanical properties of slide-ring double network hydrogels with low entangled and highly entangled secondary networks. **a** Illustrations of the cross-links and slip-links involved in the slide-ring (SR), highly entangled (HE), and low entangled (LE) networks. **b** Tensile curves of the LEN and LESRDN hydrogels are overlaid with those of the SRN, HEN, and HESRDN hydrogels for comparison. Box plots compare the **c** water content, **d** UTS, **e** maximum stretch, **f** Young's modulus (E), **g** work of fracture, and **h** fracture toughness of the LEN and LESRDN samples.

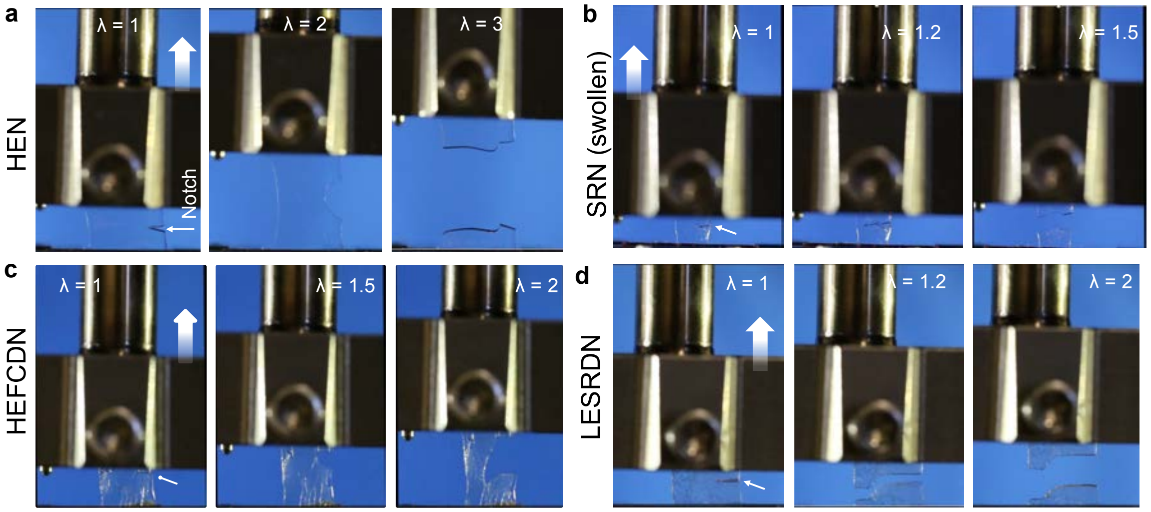

Figure S6: Photographs captured *in situ* during notch tests of fracture toughness for hydrogel samples **a** HEN, **b** SRN, **c** HEFCDN, and **d** LESRDN.

Table S1: Summary of formulations and mechanical properties of HPPR polyrotaxane-based SRN hydrogels, as-synthesized (not swollen) except where noted.

Sample	Pre-gel formulation for SRNs		Mechanical properties of SRNs				Water content (wt%)
	[HPPR] (wt%)	[DVS] (wt%)	UTS (kPa)	Stretch (λ_{max})	Stiffness (kPa)	Work of fracture (kJ m^{-3})	
SRN1	18	2.5	30±5	7.6±0.1	12±2	102±8	80±0.5
SRN2	28	2.5	55±4	4.5±0.08	30±4	122±6	70±0.5
SRN3	38	1.5	85±9	10.2±0.3	45±5	460±10	61±1
SRN4	38	2.5	240±10	11.8±0.22	60±6	1550±15	61±1
SRN5	38	4.0	205±8	4.8±0.1	105±8	510±12	60±0.5
SRN4, swollen	38	2.5	8±2	1.1±0.03	0.6±0.1	1.02±0.3	93±0.8

Table S2: Summary of formulations and mechanical properties of acrylamide-based HEN hydrogels, fully swollen

Sample	Pre-gel formulation for PAAm-HEN			Mechanical properties of HENs			
	[AAm] (M)	[MBA] (mol%) x10^{-3}	[Initiator] (mol%) x10^{-3}	UTS (kPa)	Stretch (λ_{max})	Stiffness (kPa)	Work of fracture (kJ m^{-3})
HEN1	15	1	0.5	235±8	3.5±0.1	82±4	507±8
HEN2	15	2	1	262±5	4.38±0.2	94±6	420±10
HEN3	20	1	0.5	305±10	3.97±0.1	102±8	362±25
HEN4	20	2	1	280±7	4.36±0.15	113±5	461±15
HEN5	25	0.5	0.5	302±6	3.65±0.07	130±10	432±18
HEN6	25	0.5	1	222±14	3.12±0.05	121±8	277±12
HEN7	25	0.5	1.5	303±5	4.13±0.1	85±4	435±15
HEN8	25	1	0.5	330±10	4.17±0.08	135±7	525±10
HEN9	25	1	1	287±8	4.25±0.1	128±10	466±16
HEN10	25	1	1.5	338±5	4.24±0.18	139±8	513±12
HEN11	25	2	0.5	232±15	2.63±0.2	193±5	237±20
HEN12	25	2	1	262±12	3.32±0.09	143±5	325±22
HEN13	25	2	1.5	325±10	3.58±0.1	176±6	449±11
HEN14	25	3	1.5	296±8	3.45±0.3	147±10	405±9

Table S3: Summary of formulation and mechanical properties of the double network HESRDN hydrogels, fully swollen.

Sample	Pre-gel formulation for PAAm network				Mechanical properties of HESRDN				Water content (wt%)
	[AAm] (M)	[MBA] (mol%) x10^{-3}	[Initiator] (mol%) x10^{-3}	Soak time (h)	UTS (kPa)	Stretch (λ_{max})	Stiffness (kPa)	Work of fracture (kJ m^{-3})	
HESRDN1	15	2	1.5	2	221±10	3.7±0.1	110±4	330±8	94.3±0.8
HESRDN2	20	2	1.5	2	355±11	4.9±0.1	123±4	690±13	92.7±0.8
HESRDN3	25	1	1.5	2	155±15	2.2±0.1	143±4	145±11	92.5±0.2
HESRDN4	25	2	0.5	2	343±8	3.2±0.07	155±3	403±7	94.8±0.2
HESRDN5	25	2	1.5	2	538±12	5.3±0.2	202±6	1275±18	91±0.5
HESRDN6	25	2	2	2	297±7	2.9±0.05	162±3	378±6	90±0.1
HESRDN7	25	2	1.5	0.5	340±6	3.9±0.2	145±5	547±3	95±1.0
HESRDN8	25	2	1.5	1	395±8	4.4±0.08	160±4	610±12	94.6±0.4
HESRDN9	25	2	1.5	1.5	450±10	4.9±0.1	172±10	760±8	91.3±0.8
HESRDN10	25	2	1.5	2.5	542±5	5.1±0.1	200±5	1105±15	90.7±0.9
HESRDN11	25	3	1.5	2	281±15	3.4±0.1	158±5	403±7	89.7±0.2

Table S4: Comparison of mechanical properties of various double-network hydrogels having energy dissipation mechanisms presented in recent literature.

Hydrogel	Water content (wt%)	Toughness (J m^{-2})	Work of fracture (kJ m^{-3})	Stiffness (kPa)	Reversibility (%)	Ref.
PAAm-PAMPS double network	90	1000–4500	11000	100–1000	32	[1]
Alginate-Ca^{2+}/ PAAm DN gel	90	9000	2500	29	22	[2]
Agar/HPAAm DN gel	85	1000	1023	106	20	[3]
Polymerizable rotaxane/ PAAm	60	900	270	12	97.5	[4]
Polyprotein/ PAAm	90	870	200	6	95	[5]
Na-alginate/ PVA polyelectrolyte gel	70	1450	5430	540	75	[6]
PAAm highly entangled gel	90	1450	500	150	96	[7]
Long chain PEG highly entangled gel	92	1575	854	675	99	[8]
Polyrotaxane slide-ring gel	51	3600	22000	150	>99	[9]
Hydrophilic/phobic PAAm-DVB	86	26000	18800	357	>99	[10]
Agar-PAAm DN gel	84	3960	15370	447	61	[11]
Highly entangled DN gel	89	8340	2490	180	99	[12]
HESRDN5	91	2020	1275	202	>99	This work

Table S5: Comparison of mechanical properties of control single network and double-network hydrogels.

Control Sample	UTS (kPa)	E (kPa)	Stretch (λ_{max})	Work of fracture (kJ m^{-3})	Toughness (J m^{-2})	Water content (wt%)
FCN	21±4	32±3	1.33±0.5	3.2±0.3	8±2	92.5±0.4
LEN	307±8	1015±20	1.14±0.05	40±8	109±10	66.1±0.8
HEFCDN	478±12	305±10	2.20±0.1	266±18	598±15	90.1±0.5
LESRDN	430±10	925±15	1.35±0.5	78±6	182±12	70.1±0.5

Fiber Bundle Model Framework

To quantitatively analyze the damage delocalization and predict failure strains, we employ the 1D fiber bundle model (FBM), as depicted in Figure S7A. In this model, the network consists of a series of parallel fibers connected through springs, forming a linear array. Each fiber possesses a stiffness E and has an associated failure strain $\epsilon_{\text{fail}}^{\text{fiber}}$. Additionally, the connective springs between fibers have stiffness k and a failure strain of $\epsilon_{\text{fail}}^{\text{spring}}$. If the strain of any element exceeds its failure strain, it will rupture (Fig. S7B, C), and the force it withstands will drop to zero.

The FBM is designed to capture the transition between localized and delocalized failure. In the absence of connective springs (representing the single network configuration), stress applied to a fiber localizes immediately after the rupture of the first fiber, leading to brittle failure (Figure S7D). However, introducing connective springs (representing the secondary network in double networks) allows the redistribution of stress to neighboring fibers, effectively delocalizing the damage (Figure S7D). This setup mirrors the physical behavior of the damage zone in double network hydrogels, where the secondary network acts to diffuse stress and delay fracture.

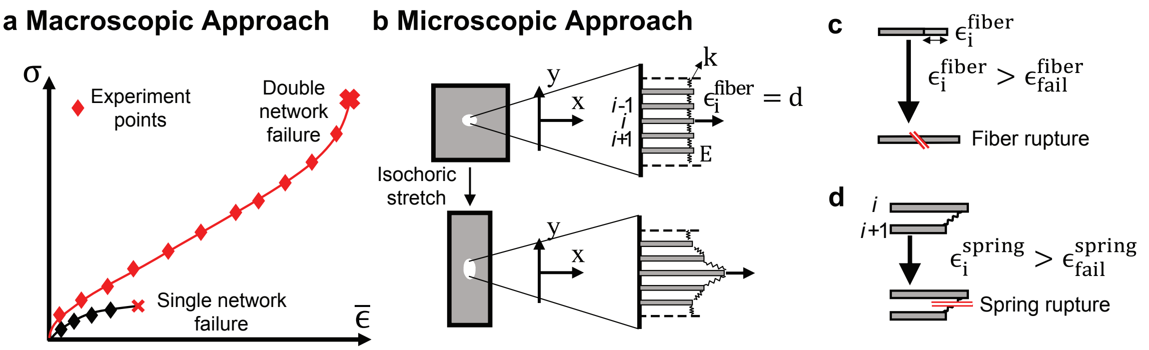

Figure S7: a) Macroscopic continuum approach is used to capture stress strain response of bulk material. b) A microscopic fiber bundle model setup is used to capture microscopic stress delocalization at damage zone. Additionally, failure criteria for both c) spring and d) fiber are visualized.

The mechanical response of the fiber bundle model can be described by the balance of forces on each fiber. The force acting on a given fiber i is given by:

$$F_i = E\epsilon_i^{\text{fiber}} + k(\epsilon_i^{\text{fiber}} - \epsilon_{i-1}^{\text{fiber}}) + k(\epsilon_i^{\text{fiber}} - \epsilon_{i+1}^{\text{fiber}}) \quad (1)$$

Here, $\epsilon_i^{\text{fiber}}$ is the strain of fiber i, and F_i is the force applied to fiber i. This equation accounts for both the intrinsic stiffness of the fiber and the stress transfer facilitated by the connective springs.

Micro-to-Macro Strain Mapping

It is important to emphasize that the fiber bundle model (FBM) employed in this work does not describe the global mechanical response of the hydrogel. Instead, it provides a mesoscopic representation of the defect zone where failure initiates. The FBM captures the local interplay between

individual fibers and springs near a damage-prone region, allowing us to model the redistribution or localization of stress and predict the strain at which this region undergoes irreversible failure.

The global stress–strain response of the hydrogel prior to failure is governed by a continuum hyperelastic constitutive model such as Gent [13] or Arruda–Boyce [14]. These models accurately describe the entropic stiffening of polymer networks under large deformations and are calibrated using macroscopic experimental data. To connect the local damage model (FBM) with the bulk continuum response, we introduce a scalar parameter α that maps the macroscopic strain $\bar{\epsilon}$ to the microscopic strain ϵ^{fiber} applied to center fiber within the damage zone:

$$\epsilon_i^{\text{fiber}} = \alpha\bar{\epsilon} \quad (2)$$

To determine α, we fit the FBM to the experimentally observed failure strain of the single network hydrogel (modeled without connective springs). Once calibrated, this scalar is held constant for all simulations, allowing direct prediction of macroscopic failure strain in double networks based on microscopic rupture events within the FBM. In this way, the FBM is used solely to compute the critical macroscopic failure strain $\bar{\epsilon}_{\text{fail}}$ of the material, while the continuum model defines the corresponding stress value through its constitutive relation. This separation of roles allows for a multiscale prediction of both the stretchability and mechanical response of double network hydrogels.

Parametric Study and Results

System Setup

To investigate the failure behavior of double network hydrogels (DNHs) using the Fiber Bundle Model (FBM), we developed a system comprising $N = 91$ fibers connected through elastic springs. The fibers are arranged in a periodic domain, where the first and last fibers ($i = 1$ and $i = 91$) are connected through periodic boundary conditions (Figure S7A). This configuration effectively mimics a continuous network, thereby minimizing edge effects and providing a more representative model of real hydrogel networks.

Each fiber in the system is characterized by its stiffness E and a failure strain $\epsilon_{\text{fail}}^{\text{fiber}}$, while each spring exhibits a stiffness k and a failure strain $\epsilon_{\text{fail}}^{\text{spring}}$. A fiber is considered disconnected (indicating crack initiation) when it fails and all its connected springs also fail. This dissociation mechanism mirrors the localized crack formation observed experimentally in double network hydrogels. Focusing on this crack initiation mode is essential, as it accurately reflects the fracture behavior of hydrogels, where localized damage zones lead to network dissociation.

Definition of the Parameter θ

To quantify the relationship between the failure strains of springs and fibers, we introduce the parameter $\theta = \epsilon_{\text{fail}}^{\text{spring}}/\epsilon_{\text{fail}}^{\text{fiber}}$. This parameter serves as a dimensionless ratio that characterizes the relative extensibility of the secondary network (springs) to the primary network (fibers). By employing θ, we efficiently capture the synergistic effects of failure strain variations between the networks.

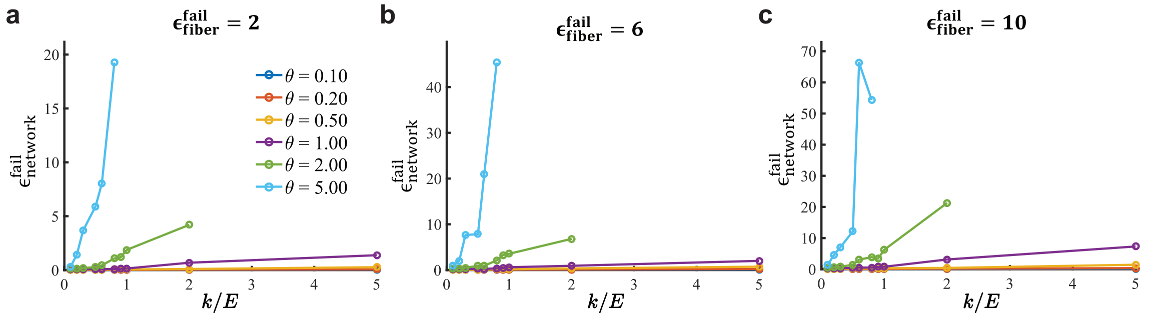

Figure S8: Fiber bundle model parametric study at fiber failure thresholds of a) 2, b) 6, and c) 10.

Parametric Study

We conducted a comprehensive parametric study to explore the effect of three critical parameters:

- **Stiffness Ratio (k/E):** The ratio of spring stiffness to fiber stiffness.
- **Fiber Failure Strain ($\epsilon_{\mathbf{fail}}^{\mathbf{fiber}}$):** The maximum strain a fiber can withstand before rupture.
- **Spring-to-Fiber Failure Strain Ratio (θ):** A dimensionless parameter representing the relative failure stretchability of the secondary network compared to the primary network.

The stiffness ratio (k/E) was varied over a range from 0.1 to 5 to capture the transition from soft to stiff secondary networks. Additionally, we investigated multiple combinations of $\epsilon_{\mathrm{fail}}^{\mathrm{fiber}}$ and $\epsilon_{\mathrm{fail}}^{\mathrm{spring}}$, quantifying the combined effects using the parameter θ.

Key Findings

Impact of k/E Ratio

The results shown in figure (S8) indicate that when the secondary springs are significantly softer than the fibers ($k/E < 1$), the network exhibits a notably higher failure strain, indicating enhanced stretchability and toughness. This can be attributed to the improved capacity of soft springs to redistribute stress throughout the network, thereby delaying localized rupture. In contrast, when $k/E \geq 1$ (stiffer springs), the system exhibits brittle behavior with lower failure strains. This suggests that stiff connective springs are inefficient in redistributing stress, leading to localized damage and rapid failure. Additionally, in double networks the secondary network is usually highly coiled up and significantly softer in compare to the primary network which is swollen [15, 16].

Comparison with Single Networks ($k = 0$)

In the special case where $k = 0$, representing a single network without any secondary elastic connections, the failure stretch of the system is significantly reduced compared to double networks. This outcome highlights the critical role of the secondary network in enhancing the mechanical resilience of the hydrogel. Without the elastic springs, the network lacks the capability to redistribute stress during deformation, resulting in a more localized and brittle failure. Single networks primarily fail through direct fiber rupture without any stress delocalization mechanism. This characteristic

leads to premature crack initiation and rapid failure propagation. In contrast, double networks, where the secondary springs provide additional stress transfer pathways, demonstrate significantly higher stretchability and toughness. This difference underlines the unique advantage of incorporating a softer, extensible secondary network, allowing for the absorption and redistribution of mechanical loads. Consequently, the double network structure effectively delays crack propagation and local failure, leading to superior mechanical performance.

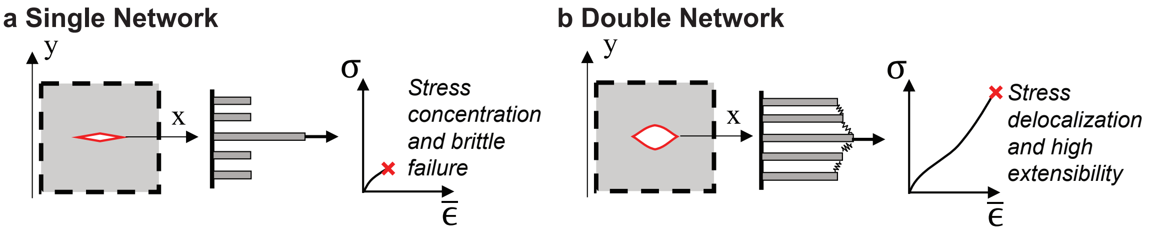

Figure S9: Conceptual representation of damage localization/delocalization mechanisms incorporated in the fiber bundle model for a) single-network and b) double-network hydrogels, where stress delocalization is introduced with connector springs.

Role of the Parameter θ

As it can be seen in figure (S8), for a fixed fiber failure strain ($\epsilon_{\text{fail}}^{\text{fiber}}$), increasing the value of θ (i.e., increasing the failure strain of the springs relative to the fibers) results in a more stretchable network. This effect arises because a higher θ implies that the secondary network remains intact for a longer period, effectively delocalizing stress during deformation. Conversely, when $\theta \leq 1$, the system exhibits brittle failure similar to a single network, as the secondary network loses its load-bearing capacity prematurely.

Synergistic Effect of k/E and θ

The most stretchable and robust network configuration occurs when:

- $k/E < 1$ (soft springs relative to fibers).
- $\theta > 1$ (springs are more extensible than fibers).

This optimal combination promotes load transfer between fibers during deformation, effectively delocalizing stress and mitigating crack propagation. Consequently, the secondary network significantly contributes to the toughness of the composite material, preventing rapid fracture by bridging damaged zones.

Effect of Primary Network Architecture on Failure Mode

While the secondary network plays a key role in delocalizing stress and preventing crack propagation, the architecture of the primary network also substantially influences the mechanical performance of double network hydrogels. In most classical DNHs, the primary network is a rigid, covalently crosslinked structure designed to fail sacrificially under deformation. Such a network exhibits brittle behavior, where individual polymer chains abruptly rupture once their local strain exceeds a critical

threshold. In the fiber bundle model, this behavior is implemented via a binary failure rule, whereby a fiber fails irreversibly when its strain exceeds $\epsilon_{\text{fail}}^{\text{fiber}}$, and its stiffness is set to zero.

To represent alternative primary networks—particularly those based on slide-ring gels or loosely crosslinked extensible polymers—we introduce a multi-stage degradation scheme for fibers. Slide-ring gels, in which cross-links are mobile and can slide along the polymer backbone, redistribute applied strain more evenly, leading to progressive softening rather than abrupt failure. In our model, this behavior is implemented through a piecewise stiffness degradation rule:

$$E_i(\epsilon_i^{\text{fiber}}) = \begin{cases} E_0, & \epsilon_i^{\text{fiber}} < \epsilon_{\text{fail}}^{\text{fiber}} \\ \frac{2}{3}E_0, & \epsilon_{\text{fail}}^{\text{fiber}} \leq \epsilon_i^{\text{fiber}} < 2\epsilon_{\text{fail}}^{\text{fiber}} \\ \frac{1}{3}E_0, & 2\epsilon_{\text{fail}}^{\text{fiber}} \leq \epsilon_i^{\text{fiber}} < 3\epsilon_{\text{fail}}^{\text{fiber}} \\ 0, & \epsilon_i^{\text{fiber}} \geq 3\epsilon_{\text{fail}}^{\text{fiber}} \end{cases} \tag{3}$$

Here, E_i is the effective stiffness of fiber i as a function of its local strain. The model allows for three progressive softening stages before complete failure, reflecting the ability of slide-ring networks to accommodate deformation through entropic mechanisms rather than immediate chain scission.

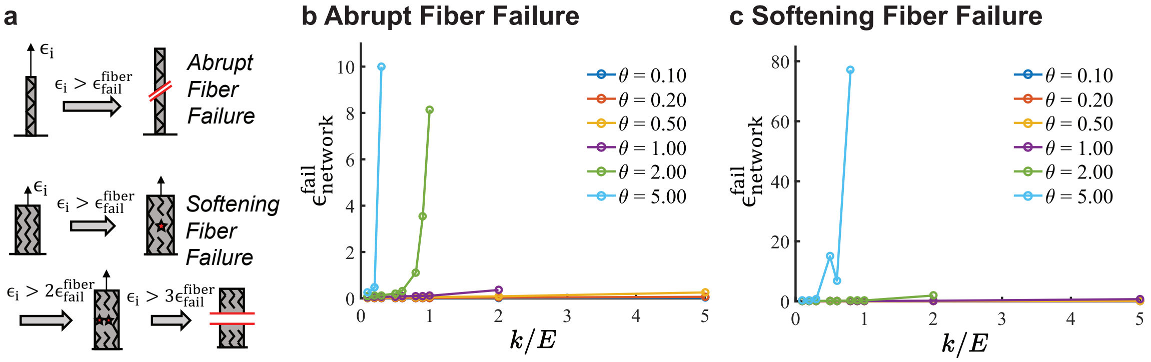

Figure S10: a) Schematic of abrupt versus softening failure styles in fibers. Softening allows gradual stiffness reduction before rupture. b) and c) show the resulting failure strain of the network across a range of k/E and $\theta = \epsilon_{\text{fail}}^{\text{spring}}/\epsilon_{\text{fail}}^{\text{fiber}}$ values, comparing the softening failure b) and abrupt rupture c) cases. The softening style, representing ductile or slide-ring networks, permits significantly higher stretchability at high θ.

By enabling this multi-stage failure mechanism in the FBM, we are able to replicate the enhanced stretchability and delayed failure observed in hydrogels with topologically adaptive or highly extensible primary networks. The combined effect of a ductile primary network and a stress-delocalizing secondary network yields a synergistic toughening strategy, expanding the design space for soft yet durable hydrogel materials.

To directly compare the effects of fiber failure mode on network-level stretchability, we conducted a parametric study under identical conditions for both abrupt (brittle) and progressive (softening) fiber degradation. As shown in Figure S10, softening fibers—representing slide-ring or ductile networks—enable dramatically higher failure strains, especially when the secondary network is both soft ($k/E < 1$) and extensible ($\theta > 1$). In contrast, networks with abrupt fiber rupture saturate at much lower failure strains, with only modest increases as θ rises. This confirms that the introduction

of gradual stiffness reduction in the primary network not only delays crack formation but also amplifies the benefit of an extensible secondary network, providing a clear design pathway for hydrogels with ultra-high stretchability.

Derivation of the Rubinstein–Panyukov Model for Uniaxial Incompressible Stretch

The Rubinstein–Panyukov (RP) model [17] is a statistical mechanics-based constitutive framework that extends classical rubber elasticity by explicitly incorporating the mechanical role of topological constraints such as chain entanglements. In this theory, the elastic free energy is decomposed into contributions from permanent network connectivity (chemical crosslinks) and from entanglement-induced constraints, the latter giving rise to a deformation-dependent amplification of stress. This structure is particularly informative for highly entangled gels, slide-ring (pulley) gels, and double-network gels, where topological constraints, nonaffine deformation, and stress redistribution play a central role. Below we outline an energy-based derivation of the engineering and true stresses under uniaxial incompressible stretch.

Step 1: Define the uniaxial incompressible deformation

For incompressible uniaxial tension:

$$\lambda_1 = \lambda, \quad \lambda_2 = \lambda_3 = \frac{1}{\sqrt{\lambda}}.$$

The first invariant of the deformation is therefore:

$$I_1 = \lambda^2 + \frac{2}{\lambda}.$$

For isotropic incompressible network models, the uniaxial stress can be written in the general form

$$P(\lambda) = M(\lambda)\left(\lambda - \lambda^{-2}\right),$$

where P is the engineering (nominal) stress and $M(\lambda)$ is a scalar prefactor determined by the strain energy.

Step 2: Energy decomposition into crosslink and entanglement contributions

In the Rubinstein–Panyukov framework, the strain energy density is written as the sum of two physically distinct parts:

$$W(\lambda) = W_c(\lambda) + W_e(\lambda),$$

where W_c represents the elastic energy associated with permanent network connectivity (chemical crosslinks), and W_e represents the contribution of topological constraints such as entanglements or cyclodextrin rings.

The crosslink contribution yields the classical Gaussian-network response with a constant modulus G_c, while the entanglement contribution introduces a deformation-dependent stiffness characterized by an entanglement modulus G_e multiplied by a dimensionless amplification factor $A(\lambda)$. Accordingly, the uniaxial stress prefactor can be written as

$$M(\lambda) = G_c + G_e A(\lambda).$$

Step 3: Construct the energy and compute engineering stress

The engineering stress is obtained from the strain energy as

$$P(\lambda) = \frac{dW}{d\lambda}.$$

To ensure consistency with the RP constitutive structure, the uniaxial energy may be written in integral form as

$$W(\lambda) = W(1) + \int_1^{\lambda} [G_c + G_e A(s)] \left(s - s^{-2}\right) ds.$$

Differentiating with respect to λ yields the engineering (nominal) stress:

$$P(\lambda) = (G_c + G_e A(\lambda)) \left(\lambda - \frac{1}{\lambda^2}\right).$$

This expression highlights the physical content of the model: G_c represents elasticity due to permanent network connectivity, while $G_e A(\lambda)$ captures the deformation-dependent contribution of entanglements and other topological constraints.

Step 4: True (Cauchy) stress

The true stress under uniaxial incompressible deformation is obtained from the engineering stress via:

$$\sigma(\lambda) = \lambda\, P(\lambda) = (G_c + G_e A(\lambda)) \left(\lambda^2 - \lambda^{-1}\right).$$

Step 5: Amplification factor for uniaxial deformation

For incompressible uniaxial stretch, Rubinstein–Panyukov theory yields a deformation-dependent amplification factor associated with the entanglement (tube-like) constraint physics. In this work, we adopt the following convenient rational approximation:

$$A(\lambda) = \frac{1}{0.74\,\lambda + 0.61\,\lambda^{-1/2} - 0.35}.$$

Substitution of this expression into the stress relations above provides closed-form formulas for both engineering and true stresses in terms of the two material parameters G_c and G_e.

This formulation is particularly useful for highly entangled gels, slide-ring gels, and double-network gels, where mechanical response arises not only from permanent connectivity but also from topological constraints and stress redistribution. By separating crosslink-like and constraint/entanglement-like contributions, the Rubinstein–Panyukov model provides a more mechanistically informative description of nonlinear elasticity than single-modulus hyperelastic models.

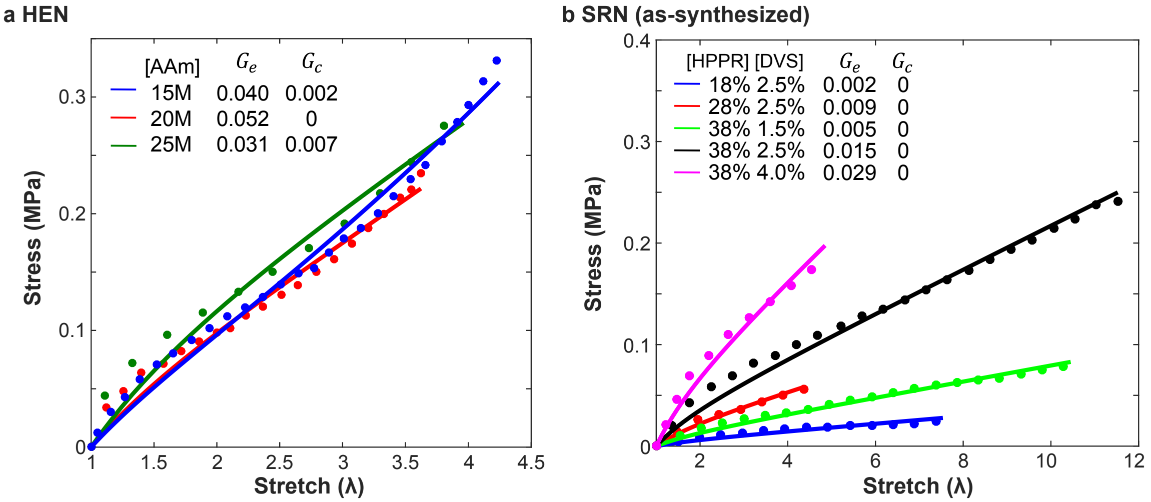

Figure S11: Rubinstein–Panyukov model fits for the component single networks a) HEN with increasing [AAm] in the pre-gel solution and b) Unswollen SRN with varying pre-gel concentrations of HPPR and DVS cross-linker.

References

[1] X. Li, J. P. Gong, Design principles for strong and tough hydrogels, *Nat. Rev. Mater.* **2024**, *9*, 6 380.

[2] J.-Y. Sun, X. Zhao, W. R. K. Illeperuma, O. Chaudhuri, K. H. Oh, D. J. Mooney, J. J. Vlassak, Z. Suo, Highly stretchable and tough hydrogels, *Nature* **2012**, *489*, 7414 133.

[3] Q. Chen, L. Zhu, H. Chen, H. Yan, L. Huang, J. Yang, J. Zheng, A novel design strategy for fully physically linked double network hydrogels with tough, fatigue resistant, and self-healing properties, *Adv. Funct. Mater.* **2015**, *25*, 10 1598.

[4] X. Xiong, Y. Chen, Z. Wang, H. Liu, M. Le, C. Lin, G. Wu, L. Wang, X. Shi, Y.-G. Jia, Y. Zhao, Polymerizable rotaxane hydrogels for three-dimensional printing fabrication of wearable sensors, *Nat. Commun.* **2023**, *14*, 1 1331.

[5] H. Lei, L. Dong, Y. Li, J. Zhang, H. Chen, J. Wu, Y. Zhang, Q. Fan, B. Xue, M. Qin, B. Chen, Y. Cao, W. Wang, Stretchable hydrogels with low hysteresis and anti-fatigue fracture based on polyprotein cross-linkers, *Nat. Commun.* **2020**, *11*, 1 4032.

[6] M. Zhang, Y. Yang, M. Li, Q. Shang, R. Xie, J. Yu, K. Shen, Y. Zhang, Y. Cheng, Toughening Double-Network Hydrogels by Polyelectrolytes, *Adv. Mater.* **2023**, *35*, 26 e2301551.

[7] J. Kim, G. Zhang, M. Shi, Z. Suo, Fracture, fatigue, and friction of polymers in which entanglements greatly outnumber cross-links, *Science* **2021**, *374*, 6564 212.

[8] G. Nian, J. Kim, X. Bao, Z. Suo, Making Highly Elastic and Tough Hydrogels from Doughs, *Adv. Mater.* **2022**, *34*, 50 e2206577.

[9] C. Liu, N. Morimoto, L. Jiang, S. Kawahara, T. Noritomi, H. Yokoyama, K. Mayumi, K. Ito, Tough hydrogels with rapid self-reinforcement, *Science* **2021**, *372*, 6546 1078.

[10] S. Tan, C. Wang, B. Yang, J. Luo, Y. Wu, Unbreakable Hydrogels with Self-Recoverable 10 200% Stretchability, *Adv. Mater.* **2022**, *34*, 40 e2206904.

[11] H. Chen, F. Yang, R. Hu, M. Zhang, B. Ren, X. Gong, J. Ma, B. Jiang, Q. Chen, J. Zheng, A comparative study of the mechanical properties of hybrid double-network hydrogels in swollen and as-prepared states, *J. Mater. Chem. B* **2016**, *4*, 35 5814.

[12] R. Zhu, D. Zhu, Z. Zheng, X. Wang, Tough double network hydrogels with rapid self-reinforcement and low hysteresis based on highly entangled networks, *Nat. Commun.* **2024**, *15*, 1 1344.

[13] A. N. Gent, A new constitutive relation for rubber, *Rubber Chem. Technol.* **1996**, *69* 59.

[14] E. M. Arruda, M. C. Boyce, A three-dimensional constitutive model for the large stretch behavior of rubber elastic materials, *J. Mech. Phys. Solids* **1993**, *41*, 2 389.

[15] Y. Chen, G. Sanoja, C. Creton, Mechanochemistry unveils stress transfer during sacrificial bond fracture of tough multiple network elastomers, *Chem. Sci.* **2021**, *12* 11098.

[16] J. Slootman, C. J. Yeh, P. Millereau, J. Comtet, C. Creton, A molecular interpretation of the toughness of multiple network elastomers at high temperature, *Proc. Natl. Acad. Sci. USA* **2022**, *119*, 13 e2116127119.

[17] M. Rubinstein, S. Panyukov, Elasticity of polymer networks, *Macromolecules* **2002**, *35*, 17 6670.
